# No evidence for environmental filtering of cavity‐nesting solitary bees and wasps by urbanization using trap nests

**DOI:** 10.1002/ece3.9360

**Published:** 2022-10-01

**Authors:** Garland Xie, Nicholas Sookhan, Kelly A. Carscadden, James Scott MacIvor

**Affiliations:** ^1^ Ecology and Evolutionary Biology University of Toronto Toronto Ontario Canada; ^2^ Ecology and Evolutionary Biology University of Colorado Boulder Boulder Colorado USA; ^3^ Department of Biological Sciences University of Toronto Scarborough Toronto Ontario Canada

**Keywords:** ecological traits, functional diversity, Hymenoptera, land cover gradient, pollinators, RLQ analysis, urban ecology

## Abstract

Spatial patterns in biodiversity are used to establish conservation priorities and ecosystem management plans. The environmental filtering of communities along urbanization gradients has been used to explain biodiversity patterns but demonstrating filtering requires precise statistical tests to link suboptimal environments at one end of a gradient to lower population sizes via ecological traits. Here, we employ a three‐part framework on observational community data to test: (I) for trait clustering (i.e., phenotypic similarities among co‐occurring species) by comparing trait diversity to null expectations, (II) if trait clustering is correlated with an urbanization graient, and (III) if species' traits relate to environmental conditions. If all criteria are met, then there is evidence that urbanization is filtering communities based on their traits. We use a community of 46 solitary cavity‐nesting bee and wasp species sampled across Toronto, a large metropolitan city, over 3 years to test these hypotheses. None of the criteria were met, so we did not have evidence for environmental filtering. We do show that certain ecological traits influence which species perform well in urban environments. For example, cellophane bees (*Hylaeus*: Colletidae) secrete their own nesting material and were overrepresented in urban areas, while native leafcutting bees (*Megachile*: Megachilidae) were most common in greener areas. For wasps, prey preference was important, with aphid‐collecting (*Psenulus* and *Passaloecus*: Crabronidae) and generalist spider‐collecting (*Trypoxylon*: Crabronidae) wasps overrepresented in urban areas and caterpillar‐ and beetle‐collecting wasps (*Euodynerus* and *Symmorphus*: Vespidae, respectively) overrepresented in greener areas. We emphasize that changes in the prevalence of different traits across urban gradients without corresponding changes in trait diversity with urbanization do not constitute environmental filtering. By applying this rigorous framework, future studies can test whether urbanization filters other nesting guilds (i.e., ground‐nesting bees and wasps) or larger communities consisting of entire taxonomic groups.

## Graphical abstract

Urbanization has no impact on cavity‐nesting bees and wasps in Toronto, Canada. We show that nesting material preference can provide novel insights. Based on this trait, there are unique assemblages across the city and this explains why there is little consensus across different cities on how urbanization affects these communities.

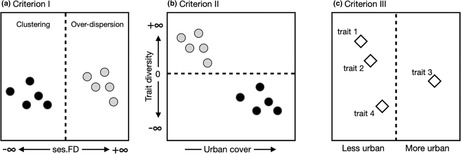

## 1 INTRODUCTION

Urbanization alters resource availability, shaping biological communities to comprise those species and traits best adapted (McKinney, 2006; Pauchard et al., 2006). In cities, increasing urbanization can lead to shifts in the taxonomic, functional, and phylogenetic structure of ecological communities (Knapp et al., 2008). Changes in community structure can then lead to broad changes in ecosystem functioning, impacting the delivery of services (e.g., pollination) in urban areas (Schwarz et al., 2017).

If impervious surface gradients exert different selective pressures on different species, cities should contain non‐random subsets of species that tolerate similar urban conditions (“environmental filtering”: Cadotte & Tucker, 2017). In other words, high levels of urbanization can act as a filter to yield a community that is composed of ecologically similar species (trees: Nock et al., 2013; bees: Hung et al., 2019; birds: Sol et al., 2020). Here, we formally define this environmental filter (by urbanization) as an ecological process that simultaneously affects key demographic parameters of a given species (i.e., survival, intrinsic growth rates, and reproduction) through habitat loss (e.g., less green space with valuable resources that affects population growth rates), which then leads to changes in species abundance (sensu Cadotte & Tucker, 2017).

**FIGURE 1:**
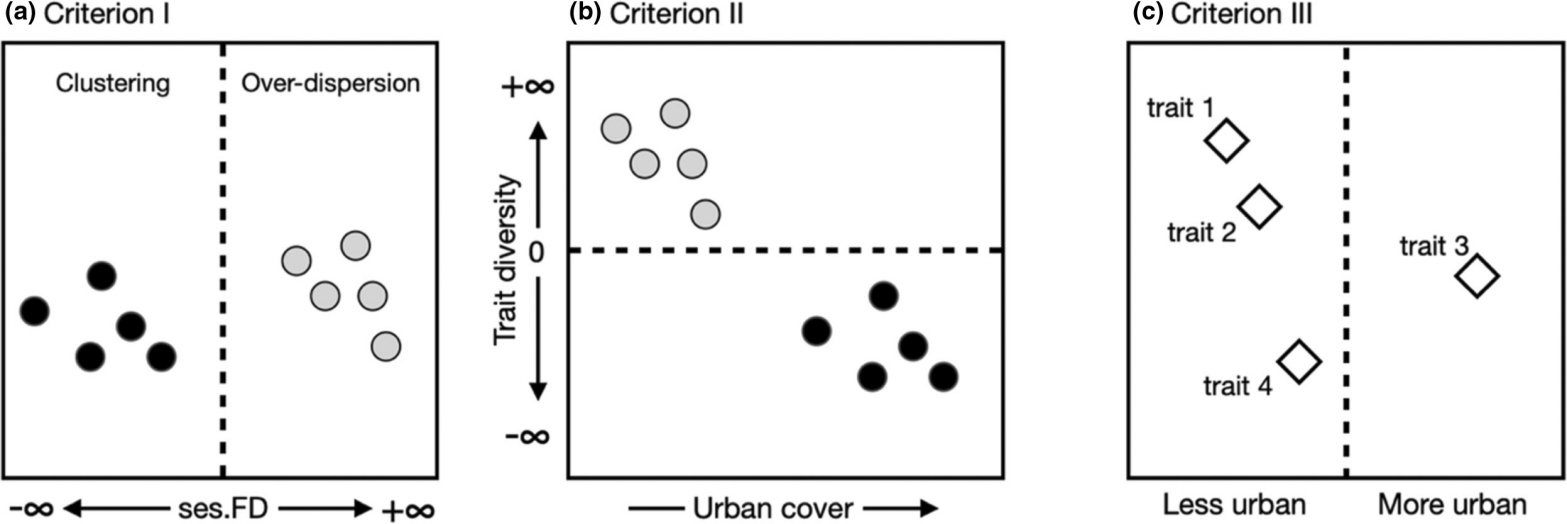
Conceptual figure of the CT framework as a robust test for environmental filtering using a trait‐based approach. (a) First, ecological communities should show significant trait clustering. (b) Second, there must be an association between trait diversity and a given environmental gradient, where the operation of filters of different strength and identity in different parts of the gradient could produce linear or more complex relationships, (c) Lastly, traits should be non‐randomly related to the environmental gradients. All three criteria must be met to substantiate environmental filtering within the sampled species pool.

If the covariance between species' growth rates and the environment influences ecological communities (Cadotte & Tucker, 2017), then observational studies of environmental filtering can shed light on how urbanization may be selecting species based on their traits. Non‐random trait clustering, where co‐occurring species share traits (or phenotypes) that are more similar than an appropriate null distribution, is often attributed to environmental filtering (but see Mayfield & Levine, 2010). Accordingly, species with traits well suited to an environment should, on average, have higher intrinsic growth rates and a competitive advantage less suited to that environment. Therefore, non‐random trait clustering suggests that certain species have higher persistence than others in a particular environment because their demographic parameters may be positively correlated with specific local environmental conditions (Figure 1). Although the detection of trait clustering can indicate a community with ecologically similar species, it is not enough evidence (by itself) to infer environmental filtering. For instance, trait clustering among different ecological communities can be observed across an urbanization gradient due to other ecological processes (e.g., herbivory, predation, and disease). In other words, non‐random clustered communities should be more frequent in highly urbanized environments (i.e., less natural habitat) compared to less urban environments (i.e., more natural habitat) (Figure 1). Finally, abundant species should possess certain traits that allow them to maximize reproductive success (i.e., maximal growth rates) in optimal environments. Therefore, traits should covary with population growth rates and urbanization. This last line of evidence to evaluate environmental filtering ensures that changes in community structure (i.e., clustering) associated with urbanization are well explained by particular traits that are responsible for an increase in population growth rates (e.g., those linked to competitive abilities).

To help mitigate challenges in linking patterns to processes, Cadotte and Tucker (2017) proposed guidelines (hereafter, the “CT framework”) to test for environmental filtering on observational community data. They recommend: (I) testing for clustering by comparing standardized effect sizes of a selected biodiversity metric (e.g., functional diversity) to appropriate null expectations, (II) showing that clustering correlates with an environmental gradient, and (III) determining that species' traits non‐randomly relate to environmental conditions (Figure 1). We apply these guidelines to test for environmental filtering in a community of solitary wild bees and wasps (as a model system) by urbanization. The use of trap nests as a standardized sampling method allows us to quantify the reproductive success of a given cavity‐nesting species by counting brood cells per nesting tube across urbanization gradients (e.g., percent impervious surface cover). It is possible to also incorporate ecological traits related to niche differences and competitive abilities (Wong et al., 2019), such as nesting material preferences and body size, respectively. Lastly, solitary bees and wasps forage near their nesting locations, implying that they are sensitive to resource preference, local availability, and environmental conditions (MacIvor, 2017; Staab et al., 2018). This model system grants the opportunity to study how urban landscapes can alter the survival and reproduction of organisms via their ecological traits, providing a link among demographic parameters (i.e., survival and reproduction), species growth rates, and trait–environment relationships as key components of the CT framework.

### 1.1 Solitary wild bees in the city

Solitary wild bees (e.g., non‐social and non‐managed bees) are important pollinators of wild and cultivated plants in many environments, including cities (Baldock et al., 2019; Lowenstein et al., 2015; Ollerton et al., 2011). Yet, there are contrasting predictions about how urbanization influences pollinators (Bartomeus et al., 2018; Wenzel et al., 2020). Urbanization may cause detrimental changes to solitary wild bee communities by replacing or fragmenting habitats (Hung et al., 2017). Alternatively, urban areas may foster bee habitat (Hall et al., 2017) as mosaics of homes, community gardens, parks, and green roofs provide heterogeneous nesting and floral resources that support a diversity of species (Aronson et al., 2017; Baldock et al., 2019; Hülsmann et al., 2015). Understanding the responses of wild bee communities to urbanization is critical for conserving their populations and managing pollination services in cities (Turo & Gardiner, 2019).

Ecological traits that characterize wild bee communities provide additional insight into how species and the pollination services they provide might respond to environmental change (Buchholz & Egerer, 2020; Ricotta & Moretti, 2011; Williams et al., 2010). For example, wild bee body size has been correlated with maximum foraging distance (Greenleaf et al., 2007). Therefore, body size could influence how bees respond to habitat fragmentation (Bommarco et al., 2010); larger bees may be able to forage further to locate nesting and floral resources (but see Biedermann, 2003). In turn, an ability to reach habitat isolated in parks and gardens may make large‐bodied bees critical for urban pollination services (Palma et al., 2015). Urban environments provide longer flowering periods with more non‐native plant species, as well as increased floral availability toward the end of the growing season (Dallimer et al., 2016; Fisogni et al., 2020). This consistency in plant availability may enable longer access to resources for generalist species in cities. Further, species of non‐native wild bees are overrepresented in surveys from urban areas compared to natural areas (Fitch et al., 2019; Matteson et al., 2008; Normandin et al., 2017; Wilson & Jamieson, 2019) because they can potentially exploit non‐native flowering plants and outcompete native bees for similar nesting resources (Russo, 2016). Nesting materials preferred by wild bee species may also limit both their geographic location and abundance across urban landscapes. Specifically, certain species should have lower population densities in areas with fewer resources (Fisher & Owens, 2004). For example, the leafcutter bee *Megachile pugnata* Say (Megachilidae) has been shown to collect leaves from fewer plant species than other common leafcutter bee species (MacIvor, 2016a), and may not tolerate highly impervious areas where these plants are not found. Lastly, wild bees that nest in cavities above ground (e.g., in wood and built infrastructure; MacIvor, 2017) could be less limited by urbanization than ground‐nesting bees, which are excluded where sealed surfaces cover porous soils and other groundcovers (Cane et al., 2006; Pereira et al., 2020). Evaluating cavity‐nesting bees should thus provide a robust test of environmental filtering by urbanization.

### 1.2 The importance of solitary wasps in cities

Although wasps and bees are closely related (Sann et al., 2018), wasps prey on arthropods rather than collecting pollen and nectar from flowers. Yet, wasps and bees share similar nesting locations (e.g., above‐ground cavities in wood or plant stems or built infrastructure) and some nesting material preferences (see Krombein, 1967; O'Neill, 2001). Despite the critical role of wasps as predators to regulate populations of abundant and/or pest arthropods (Careless et al., 2014; Grissell, 2010), compared to solitary bees, there has been relatively little research on solitary wasp communities (Sumner et al., 2018). Further, wasp persistence in urban areas is threatened by lack of information, public disdain (Shipley & Bixler, 2017), as well as physical removal of wasps from urban areas (Fowler, 1983). An 80‐year land‐use change study showed that wasp species richness declined at the same rates as bees' through habitat loss in the United Kingdom (Senapathi et al., 2015). However, not all arthropod groups respond the same way to urbanization in terms of abundance, diversity, and ecological traits (Fenoglio et al., 2020). For example, many prey species sought by solitary wasp species are abundant in urban landscapes and might sustain urban wasp populations (e.g., aphids; Rocha et al., 2018). Therefore, it is not clear whether solitary bee and wasp communities will show concordant or discordant responses to urbanization.

### 1.3 Using the CT framework to test for environmental filtering

Our study tests whether environmental filtering structures communities of cavity‐nesting solitary bees and wasps using the three criteria of the CT framework. Urbanization has been shown to negatively impact many species as impervious surfaces replace natural habitats and resources required for survival and reproduction. We hypothesize that environmental filtering by urbanization occurs, and we predict that this community will satisfy each of the three filtering criteria requirements: first, there is significant trait clustering in sites with a high percentage of impervious cover (Criterion I). Second, there is a positive relationship between clustering and urbanization (Criterion II). Third, traits are non‐randomly related to environmental conditions (Criterion III). Lastly, we aimed to identify whether certain solitary cavity‐nesting bee or wasp ecological traits are over‐ or underrepresented in distinct urban green space types, or associated with urbanization, to interpret how and where practitioners could prioritize conservation through management at the habitat (e.g., vegetative management) and the landscape scale (e.g., promote a network of urban green spaces).

## 2 MATERIALS AND METHODS

### 2.1 Sampling

In this study, we surveyed solitary cavity‐nesting bees and wasps, which commonly nest in beetle‐bored holes in logs, hollow plant stems, as well as in built infrastructure (e.g., nail or drill holes in mortar, brick or wood), and intentional, human‐made structures such as trap nests (MacIvor, 2017). Trap nests are bundled nesting holes (e.g., plant stems or drilled holes in wood) that are commonly deployed to support these taxa (Staab et al., 2018). We installed trap nests at 200 sites (one per site) from 2011 to 2013 across the city of Toronto (Canada) and the surrounding region. Each site represented one of four common types of urban green spaces in Toronto: community garden, home garden, public park, or green roof (Figure S1). Trap nests consisted of a 30 cm section of PVC pipe with 30 cardboard nesting tubes (15 cm length) inserted, each of one of three diameters (10 of each: 7.6, 5.5, and 3.4 mm), and set up from April to October each year. We removed brood cells from each nesting tube and stored it (at 4°C) from October to March, then incubated at 26°C and 65% humidity. We identified adult bees and wasps to species level using dichotomous keys and identification resources (listed in Tables S1 and S2), compared specimens to synoptic bee and wasp collections at the last author's institution, and in a few cases, corroborated identifications with DNA barcoding completed at the Centre for Biodiversity Genomics at the University of Guelph. A total of 31 cavity‐nesting bee species and 20 cavity‐nesting wasp species were identified across all sites and sampling seasons. Cleptoparasitic bees and parasitoid wasps recovered from trap nests occupied by cavity‐nesting bees and wasps were not included in subsequent analyses. Representative bees and wasps from these surveys are curated in the collections of the Biodiversity of Urban Green Spaces (“BUGS”) lab at the University of Toronto Scarborough.

### 2.2 Landscape variables

**FIGURE 2:**
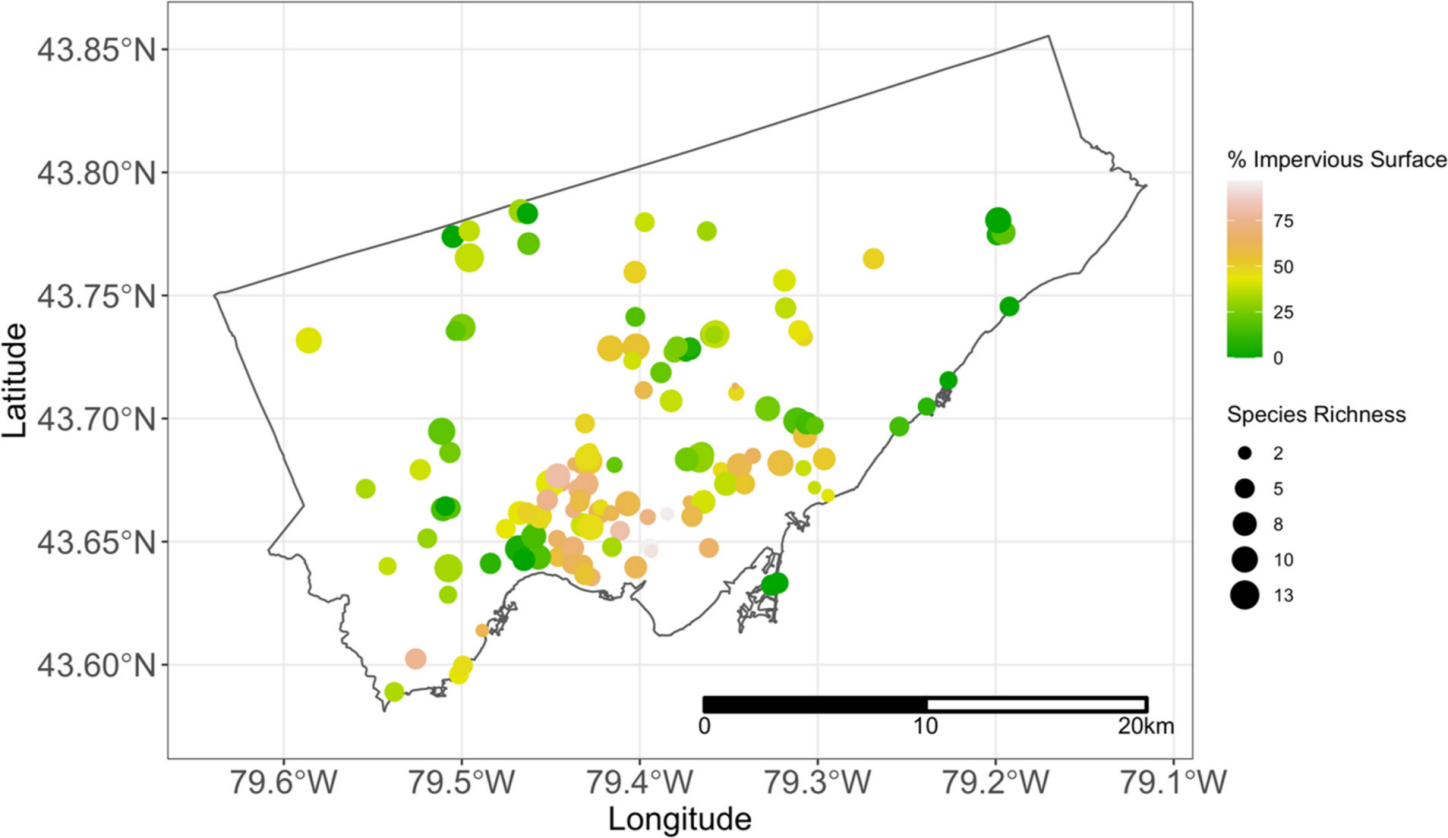
Map of an urbanization (impervious surface) gradient of all sampled sites at the 250 m buffer radii within Toronto, Canada. The size of the circles represents different levels of species richness. The source material for the regional municipal boundary is from the City of Toronto Open Data Portal. The geographic coordinate system is WGS84 (latitude and longitude).

Urbanization encapsulates many different anthropogenic activities, so it is best quantified as multiple‐component gradients (Moll et al., 2019). Here, we quantified the percent land cover class from three components—impervious cover, open green cover, and closed green cover—from the 2008 Forest and Land Cover dataset (0.6 m raster pixel resolution; Pinto, 2008). We estimated urbanization (“impervious surface cover”) as the sum of the proportion of buildings, roads, and other paved surfaces. To better resolve the geographic scale at which environmental gradients impact solitary bees and wasps, we quantified impervious surface cover at two different spatial scales (250 m radii: range [0%–97%]; 500 m radii: range [0%–93%]; see Data Availability Statement). Both spatial scales reflect those of previous studies that examine “realistic” maximum flight ranges of solitary bee species (Gathmann & Tscharntke, 2002; Greenleaf et al., 2007; Zurbuchen et al., 2010). Also, other studies have shown that local environmental factors at the 250 m scale influence bee communities (Hofmann et al., 2020; Steffan‐Dewenter et al., 2002; Williams & Winfree, 2013). The range of percent urban cover also varies across different urban green space types and spatial scales (i.e., community garden: 18%–72% [250 m], 21%–77% [500 m]; home garden: 10%–81% [250 m], 18%–72% [500 m]; public park: 0%–76% [250 m], 0%–73% [500 m]; and green roof: 0%–97% [250 m], 22%–93% [500 m]). We also calculated the percent open and closed green space cover for each site and scale. Here, we removed the “water” land cover class from this analysis. We could not calculate landscape variables for sites that were not completely contained within the raster dataset and so 192 sites were used for subsequent analyses at the 250 and 500 m scale (Figures 2 and S2). We calculated landscape variables for all sites using R version 4.1.1 (R Core Team, 2021; all further analyses were completed using this software).

### 2.3 Bee and wasp traits

We chose seven ecological traits based on their potential to influence the response of solitary cavity‐nesting wild bees and wasps to environmental variation in urban landscapes and their contribution to pollination and arthropod predation services (Buchholz & Egerer, 2020). The first six traits were determined from primary literature sources: native status (i.e., native or non‐native), primary diet type (i.e., pollen for bees and preferred prey item for wasps), diet specialization (i.e., the taxonomic resolution of the diet preference), trophic rank (i.e., herbivore, feeds on herbivore, and feeds on carnivore), nesting material preference (i.e., the materials collected), and the number of nesting material types collected (see Tables S1 and S2 for details). Lastly, we measured bee and wasp body sizes directly from our sampled populations. Body size was determined using mean female intertegular span (mm), measured as the linear distance between the wing tegulae, across the thorax (Cane, 1987), and from a minimum of five individual females per species. Due to the insufficient numbers of females in our study needed to determine intertegular span, we used the values for four bee species (i.e., *Anthophora terminalis*, *Hoplitis producta*, *Hylaeus hyalinatus*, and *Hylaeus punctatus*) but no value was available for two wasp (i.e., *Passaloecus monilicornis* and *Symmorphus bifasciatus*) and two bee species (i.e., *Heriades variolosa* and *Hoplitis spoliate*), which were removed from the analysis (Table S1). Further, one bee species *Hylaeus punctatus* (Brullé) was found at a single site outside the city boundary and as a result, was excluded from the analysis. Thus, we had a total of 46 species, consisting of bees (*N* = 28) and wasps (*N* = 18), with a complete set of sampled traits for subsequent analyses.

### 2.4 Statistical analyses

#### 2.4.1 Criterion I: Clustering of phenotypes in sampled communities

The first guideline of the CT framework requires evidence of significant clustering of ecological traits in species communities. To determine if this criterion is met, we first measured the abundance (i.e., number of completed brood cells, including parasitized/diseased individuals) of all species from each site that were sampled across 3 consecutive years (i.e., 2011, 2012, 2013). We chose this measure as it represents the total abundance of a given species in a particular environment, which should reflect the combined effects of local competition and environmental filtering. A prior exploratory analysis showed no high interannual variation in the distribution of the number of completed brood cells (Figure S3) and species richness (Figure S4) for both bees and wasps across all sites, so we combined raw abundance data for all three sampled years into a single‐community data matrix (Figures S3 and S4). We retained data for 136 sites for our Criterion I analysis after removing sites with insufficient data (48 sites sampled in <3 years, four sites that remained uncolonized in all years, and four sites containing single species, since a minimum of two species are needed to assess phenotypic clustering).

To satisfy CT Criterion I, we calculated functional alpha diversity within communities as the standardized effect size of the abundance weighted mean pairwise functional distance (hereafter “ses.MFD”). Here, we used the “mpd” function from the *picante* package with a functional distance matrix (Kembel et al., 2010). Gower's distance accommodates both continuous and categorical variables and was used to construct the functional distance matrix from the selected seven traits (Gower, 1971).

To test for the effects of environmental filtering, we compared the observed MFD values with simulated communities by randomizing the community data matrix abundances within species, which maintains species occurrence frequency (Kembel et al., 2010). We calculated ses.MFD as:

(1) $$\mathrm{ses}.\mathrm{MFD}=\frac{\left({\mathrm{MFD}}_{\mathrm{obs}}-{\mathrm{MFD}}_{\text{null}}\right)}{{\mathrm{SD}}_{\text{null}}}$$

where MFD_obs_ is the observed value (mean pairwise functional distance), MFD_null_ is the mean of the simulated values from the 4999 randomized communities, and SD_null_ is the standardized deviation of those simulated values (Webb et al., 2002). All alpha levels for null hypothesis testing (*H*_0_: ses.MFD = 0; two‐tailed tests to account for both clustering and overdispersion) were set to 0.05 throughout.

A trait randomization approach, by which trait values are randomly shuffled within dominant and rare species, can offer a similar distribution of expected values to that of a community matrix‐based approach. The outcomes are similar because a trait diversity index (i.e., ses.MFD) can be weighted by species abundances, and thus, it is impossible to tease apart weaker competitive exclusion and environmental filtering when the same trait is connected to both mechanisms (see Götzenberger et al., 2016, for more detail). Therefore, we only include the community matrix‐based approach in our analysis.

#### 2.4.2 Criterion II: Association among clustering, urbanization, and urban green space type

The second criterion requires that an environmental gradient be associated with the degree of clustering within communities. To determine if this criterion was met, we regressed ses.MFD against three different urbanization gradients (i.e., percent impervious surface, percent closed green cover, and percent open green cover) at the 250 and 500 m buffer radii, and urban green space (UGS) type as a categorical variable (i.e., community garden [*n* = 13], home garden [*n* = 67], public park [*n* = 42], and green roof [*n* = 14]). We removed percent closed green cover from this analysis because it was highly collinear with the impervious surface (Figures S5 and S6; Tables S3 and S4). Therefore, we completed two separate linear regression models with a two‐tailed test for this analysis for both spatial scales as follows: ses.MFD ~ percent open green cover + percent impervious surface + UGS type.

We also did pairwise comparisons (i.e., estimated marginal means) between urban green space types, while accounting for unbalanced sample sizes, using the “emmeans” R package (Lenth, 2020). We did not include spatial covariate structures in our regression models since model residuals were not spatially autocorrelated (250 m: observed Moran's *I* = −3.57 × 10^−3^, *p* = .719; 500 m: observed Moran's *I* = −4.46 × 10^−3^, *p* = .77; Dale & Fortin, 2014).

#### 2.4.3 Criterion III: Multiple traits covary with urbanization

We used RLQ analysis (Dolédec et al., 1996) to test for covariance between different environmental variables (R‐table) and species trait values (Q‐table), constrained by species abundance (L‐table) for each spatial scale. Here, we had a total of 140 sites for this analysis (including those occupied by a single species). This multivariate test determines if certain species or traits are associated with environmental conditions. Our R‐table includes the proportion of each land cover (i.e., percent open green cover, percent closed green cover, and percent impervious surface) across the entire study region. The Q‐table characterized 46 bee and wasp species based on seven ecological traits (i.e., body size, origin, nesting material type, number of nesting material types, diet, specialization, and trophic rank; see Table S1 for bees and Table S2 for wasps). At each spatial scale, we performed separate analyses on each table (R, Q, and L) for the RLQ analysis. First, we conducted a principal component analysis (PCA) on the R (environmental) table. We analyzed the Q (trait) table with an extended PCA technique to account for a mix of multi‐state discrete and continuous variables (Hill & Smith, 1976). We then calculated column and row weights of the R‐ and Q‐tables by using the site and species scores from correspondence analysis on the L (abundance) table. Second, we performed the RLQ analysis, which links all three tables to produce a simultaneous ordination of environment, traits, and species composition. We conducted all ordinations using the *ade4* package (Dray & Dufour, 2007).

In accordance with CT Criterion III, we tested species–trait–environment associations (Q → R) using a sequential test approach as a global test of the significance of the RLQ analysis, outlined in ter Braak et al. (2012) to account for both inflated type I errors and a lack of a common unit of observation between all three tables (R, Q, and L). Specifically, we can test the statistical significance of two separated links: Model 2, the link between the community data matrix and the traits by permuting columns (L → Q), and Model 4, the link between the community data matrix and the environmental variables by permuting entire rows (L → R). If we reject the null hypothesis for both links (ter Braak et al., 2012), then we have strong evidence that there are trait–environment associations between ecological traits and the percent impervious surface gradient. If we fail to reject both null hypotheses, then this result precludes continuing to the fourth‐corner analysis of pairwise trait–environment associations. Here, we set the number of permutations for each test to be 49,999 runs.

## 3 RESULTS

### 3.1 Criterion I: Clustering of phenotypes in sampled communities

**FIGURE 3:**
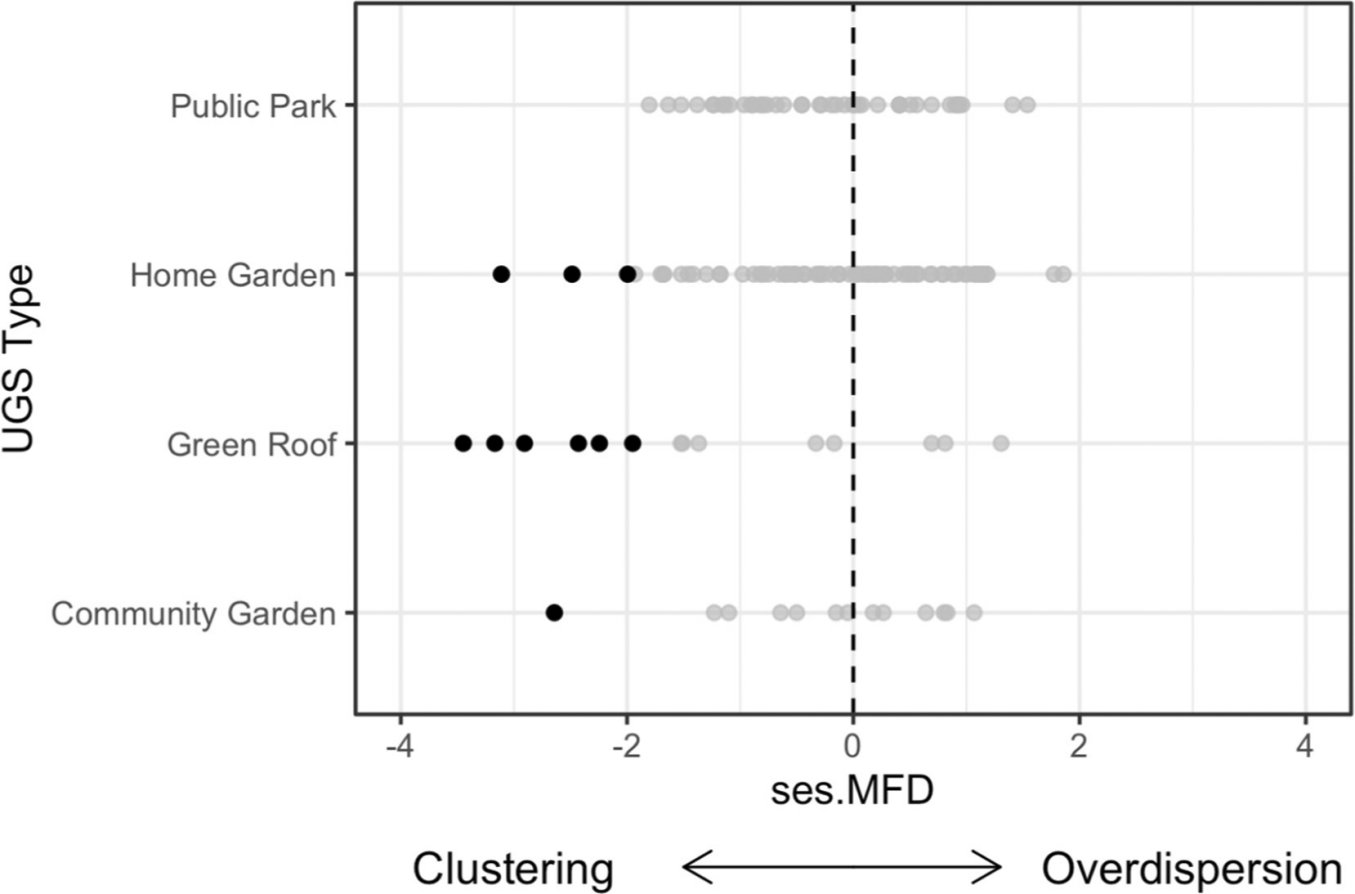
A scatterplot of standardized mean pairwise functional distances (ses.MFD) and urban green space (UGS) type. Here, 10 of 78 sites showed significant clustering (ses.MFD < 0; *p* < .05) as indicated by the highlighted black dots (i.e., six green roofs, three home gardens, and one community garden).

Trap nests contained six species on average (range: 1–13 species). Of the 136 communities included in this analysis, 77 showed trait clustering (ses.MFD < 0) but only a small fraction (*N* = 10; six green roofs, three home garden, and one community garden) were significantly clustered (*p* < .05; Figure 3). Most communities in the study area were randomly assembled from the regional species pool (*n* = 126), and none had significant trait overdispersion (ses.MFD > 0).

### 3.2 Criterion II: No relationship between clustering and urbanization, but there is significant clustering on green roofs

**FIGURE 4:**
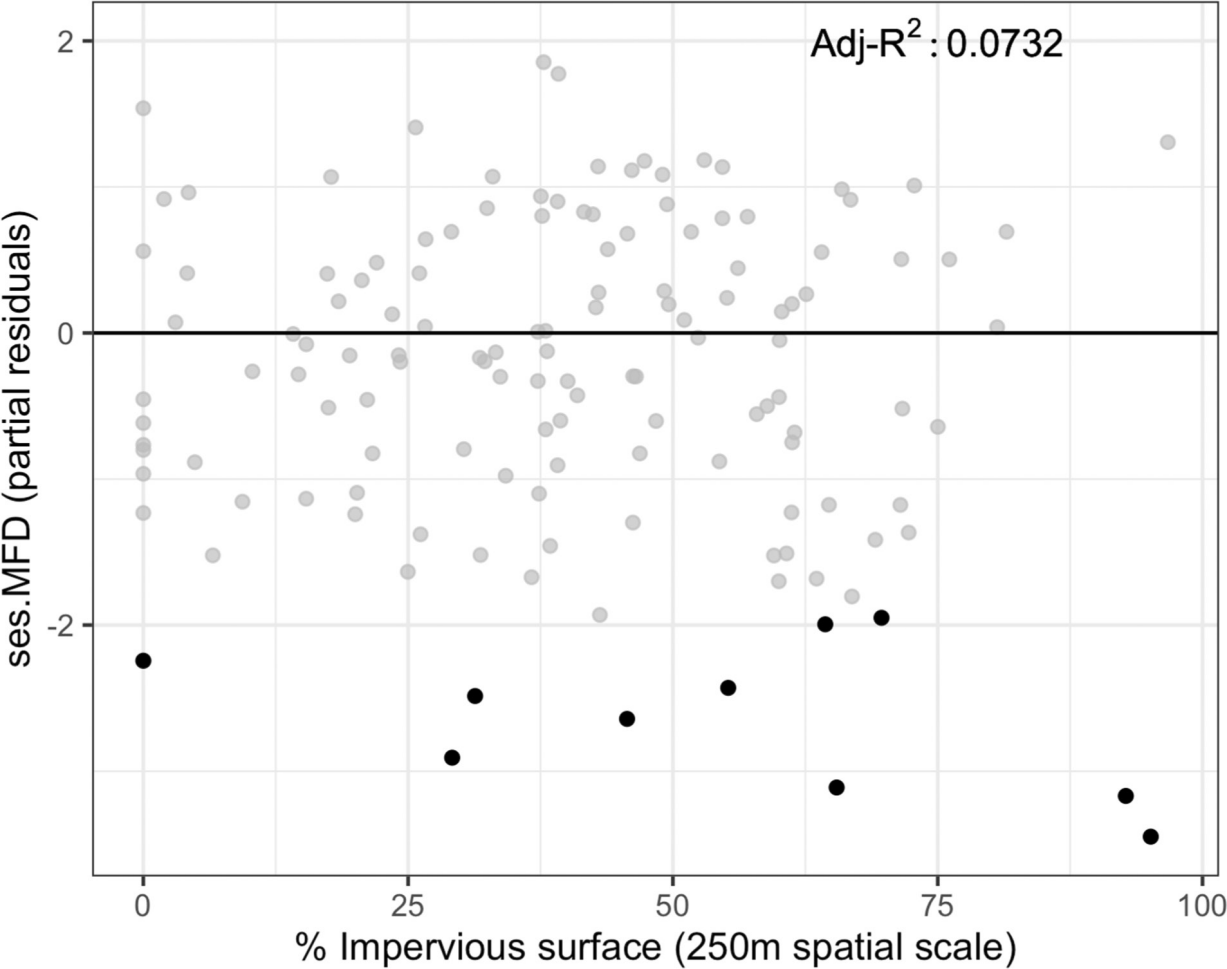
A scatterplot of percent impervious surface and standardized mean pairwise functional distances (ses.MFD) at the 250 m spatial scale. Highlighted black dots represent sites that meet CT criterion I (*n* = 10—communities with significant functional clustering [*p* < .05; see Figure 3]).

After accounting for the percent open green space cover and UGS type (Table S4), there was no relationship between the percent impervious surface and ses.MFD at the 250 m scale (*β*_imp_ = −0.003, *df* = 130, *t* = −0.58, *p*‐value = .56; Figure 4). Similar patterns were also found at the 500 m scale (Figure S7; Tables S5 and S6). Together, the results suggest that there is no relationship between changes in trait clustering for cavity‐nesting bees and wasps and the percent impervious surface. In addition, there was significant trait clustering only for green roofs when compared to community garden (*β* = 1.23, SE = 0.43, *t*.ratio = 2.84, *p* = .03), home gardens (*β* = 1.18, SE = 0.31, *t*.ratio = 3.71, *p* < .01), and public parks (*β* = 1.05, SE = 0.38, *t*.ratio = 2.78, *p* = .03) in the 250 m scale. Similar patterns for such pairwise comparisons were also found at the 500 m scale (data not shown).

### 3.3 Criterion III: Traits are randomly related to urbanization

**FIGURE 5:**
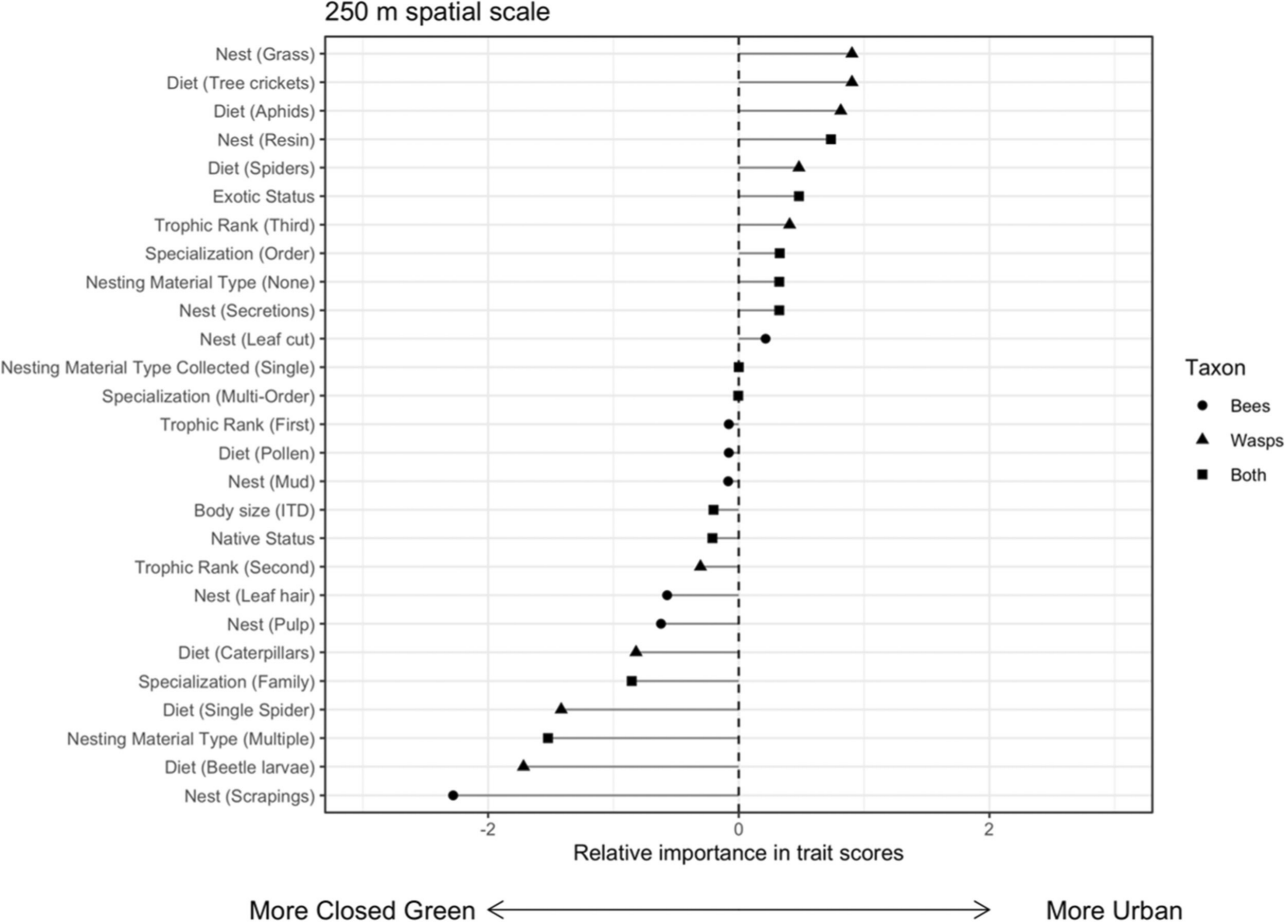
The relative importance of trait scores from RLQ axis 1 for the 250 m spatial scale. Negative score values indicate traits that are correlated with more closed green cover while positive score values indicate traits that are more correlated with impervious surfaces.

**FIGURE 6:**
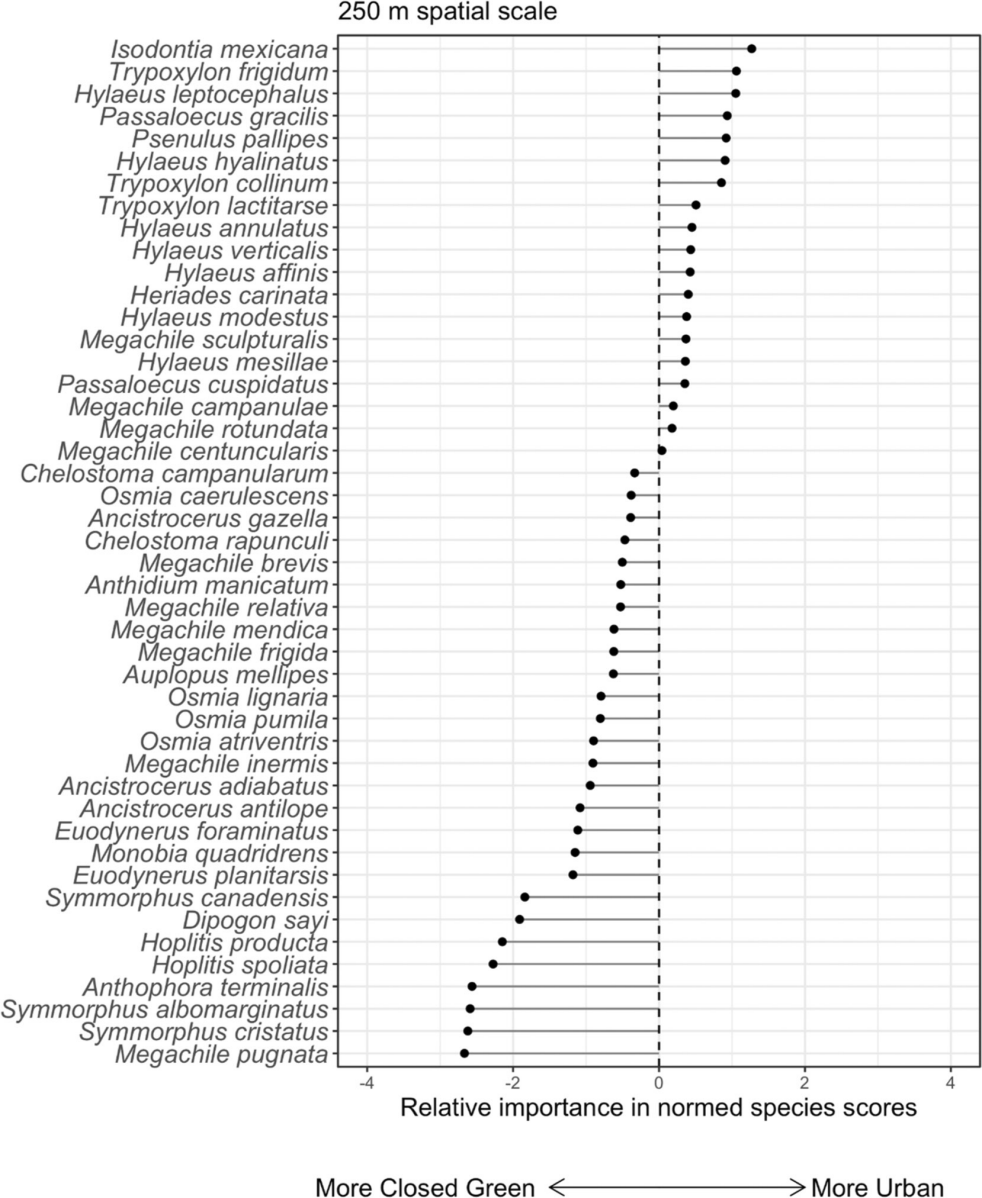
The relative importance of normed species scores from RLQ axis 1 for the 250 m spatial scale. Negative score values indicate species that inhabit sites with more closed green cover while positive score values indicate species that inhabit sites with more impervious surfaces.

Environmental conditions (i.e., percent closed green cover, percent open green cover, and impervious surface) did not influence the distribution of ecological traits at either spatial scale (Model 2—250 m: *p* = .91; 500 m: *p* = .87). In addition, the seven ecological traits were not associated with the composition of species assemblages found in our sampled sites within the three environmental variables (Model 4—250 m: *p* = .99; 500 m: *p* = .99). Despite a covariance among traits, species abundance, and environmental conditions across different spatial scales (Figures 5, 6 and S8–S12), there is no strong evidence of a species–trait–environment association among our sampled communities.

## 4 DISCUSSION

In our study, we applied the CT (Cadotte & Tucker, 2017) framework to test for environmental filtering in a community of solitary cavity‐nesting wild bees and wasps by urbanization. Using three independent criteria, we cannot conclude that urbanization acts as a filter on these bee and wasp communities based on their ecological traits. First, only a small fraction of our sampled communities (10 of 136) showed significant clustering of phenotypes, thereby failing Criterion I. Second, we found no relationship between clustering and urbanization, failing Criterion II. Third and finally, we found no evidence of species–trait–environment association, which suggests that traits are randomly related to the environment, failing Criterion III. Our study shows that trait diversity is robust to urbanization gradients within cities, suggesting that the fine‐scale patchwork of habitats in cities may be sufficient to maintain diverse communities in urban areas, at least for highly mobile cavity‐nesting bee and wasp species using trap nests.

### 4.1 Criterion I: A small fraction of communities showed significant trait clustering, specifically in green roofs

Only 10 sites of 136 were significantly clustered, 60% of which were green roofs (Figure 3). Green roofs are increasingly common in cities around the world, and especially in Toronto where there is a mandatory bylaw for select new building types, a construction standard, and an incentive program (City of Toronto, 2021). However, green roofs are isolated from ground level and more exposed to sun, wind, and drought, impacting contributions to urban biodiversity (Williams et al., 2014). Among the green roofs surveyed, MacIvor (2016b) showed that bee and wasp diversity in trap nests declined with building height. Further, green roofs are mostly designed with non‐native and horticultural plant species (e.g., Sedum) and could be more attractive to non‐native bees (MacIvor et al., 2015). It is possible that due to the harsh conditions present on green roofs that exclude some species, as well as higher competition for available nesting tubes in trap nests at ground level by other bee and wasp species, trap nests installed on green roofs might act as refugia for non‐native cavity‐nesting species in the city. This observation is limited by our surveys being exclusively from trap nests, and broader sampling procedures are needed to evaluate these relationships.

### 4.2 Criterion II: There is no impact of urbanization on cavity‐nesting bees and wasp diversity

We found no relationship between clustering and urbanization (Figures 4 and S7). These findings lend support to previous studies that suggest urbanization level does not negatively impact cavity‐nesting bees (Banaszak‐Cibicka & Żmihorski, 2012; Cardoso & Gonçalves, 2018; Fortel et al., 2014) and wasps (Zanette et al., 2005). The maintenance of diversity across urbanization gradients may be because there are opportunities for bees and wasps to seek novel nesting opportunities in infrastructures such as cavities in walls, eaves, and roofs (but see Guenat et al., 2019). For example, *Isodontia mexicana* (Saussure) is regularly found nesting in crevices on the exterior of houses (e.g., the windowsill); the native wasp is tolerant of human activity and has even established in several European countries (Polidori et al., 2018). In another example, *Megachile rotundata* (Fabricius) nest in an array of materials, from car radiators (Sheffield, 2017) to abandoned invasive paper wasp nests sheltered under building roof awnings (MacIvor, 2021). Other bee and wasp species might also benefit from these fine‐scale environmental conditions which are difficult to quantify but could represent resources that shape diversity urbanization patterns. While we did not measure such local factors in our study, we can speculate on the mechanisms by which these resources could promote wild bee and wasp diversity in urban areas. For example, sun exposure reduces thermal constraints for bees (Willmer & Stone, 2004), which could affect the choice of nesting habitats; in an experimental study, *Osmia bicornis* (Linnaeus) (Family: Megachilidae) avoided tree‐shaded (less urban) areas and instead strongly preferred balconies, backyards, and parks with full sun exposure (Everaars et al., 2011). Future research that incorporates both landscape and local factors will further clarify how urbanization affects the functional community structure of cavity‐nesting species in trap nests within and across different cities.

### 4.3 Criteria III: There is no relationship among traits, species abundance, and landscape‐level environmental conditions

The RLQ analysis did not support Criteria III (Figures 5 and S11), implying that even individual traits uniquely associated with solitary cavity‐nesting bees and wasps (e.g., nesting material preferences) are not linked to how community structure is affected by changes in impervious surface cover. This finding is consistent with a previous study that shows the abundance of cavity‐nesting species responds weakly to land‐use gradients via their ecological traits (Palma et al., 2015). A lack of strong landscape–trait associations could be due to the choice of impervious surface cover as a coarse landscape variable, leading to no common responses of individual traits across urbanization gradients among different cavity‐nesting species (Bartomeus et al., 2018; Moretti et al., 2021). A more predictive approach could be to test whether changes in finer‐scale environmental measures (e.g., suitable soil as nesting substrate, microclimates, and resource of preferred prey) affect species abundance, and then link this relationship with shifts in ecological traits. Partnering with community science programs could make a finer‐scale study possible, if city residents quantify local microhabitats and contribute naturalist observations. For example, community scientists provided photos demonstrating an affinity for mowed or disturbed grass by two closely related ground‐nesting bee species with overlapping flight seasons (i.e., *Adrena fulva* and *Adrena cineraria*; Maher et al., 2019).

### 4.4 Insights into the importance of nesting material and prey preference

We do make some observations in our study based on our RLQ results (Figure 5) acting as an exploratory analysis, which hints at certain ecological traits (Table S1) that could influence which species perform well in urban environments. Of course, more detailed work is needed to precisely identify the nesting and foraging resources of these species to integrate into future analyses. Nevertheless, we observed that cellophane bees (*Hylaeus*: Colletidae) secrete their own nesting material and tend to be overrepresented in urban areas, while native leafcutting bees (*Megachile*: Megachilidae) were found to be common in greener areas. For wasps, prey preference was important, with aphid‐collecting (*Psenulus* and *Passaloecus*: Crabronidae) and generalist spider‐collecting (*Trypoxylon*: Crabronidae) wasps well represented in urban areas and caterpillar‐ and beetle‐collecting wasps (*Euodynerus* and *Symmorphus*: Vespidae, respectively) overrepresented in greener areas (Figure 6).

## 5 CONCLUSION

Robust tests for environmental filtering of ecological communities by urbanization using all three criteria of the CT framework are critical for understanding the impacts of urban development on biodiversity. We did not find strong evidence for environmental filtering of solitary cavity‐nesting bees and wasps using trap nests, but we demonstrate the utility of this approach and highlight ecological traits that can provide novel and applied insight for urban conservation and planning. Different ends of the urbanization spectrum within a city offer opportunities, and support assemblages of cavity‐nesting bees and wasps. Cities around the world vary in historic and current green space compositions and configurations, which influence the habitat and resources available to cavity‐nesting bees and wasps. The landscape–trait associations we identify here likely help explain why there remains little consensus across different cities on whether urbanization has a negative, positive, or no effect on these invertebrate communities.

## AUTHOR CONTRIBUTIONS

**Garland Xie:** Conceptualization (equal); formal analysis (lead); project administration (equal); software (lead); supervision (lead); visualization (lead); writing – original draft (equal); writing – review and editing (equal). **Nicholas Sookhan:** Formal analysis (supporting); software (supporting); writing – review and editing (equal). **Kelly A. Carscadden:** Conceptualization (equal); formal analysis (supporting); writing – review and editing (equal). **James Scott MacIvor:** Conceptualization (equal); data curation (lead); funding acquisition (lead); investigation (lead); methodology (lead); project administration (equal); writing – original draft (equal); writing – review and editing (equal).

## CONFLICT OF INTEREST

The authors declare that they have no conflicts of interest.

## Supporting information


Appendix S1

## Data Availability

All data and scripts relevant for reproducing the statistical analyses are available on Github (https://github.com/garlandxie/JAE_env_filt). In addition, publicly available data and scripts are archived on DRYAD (https://doi.org/10.5061/dryad.z8w9ghxg0), with the exception of sensitive geographic coordinates (please contact the corresponding author to request these data). Please note that a subset of the data has been published in a previous journal article (https://doi.org/10.1002/ece3.7537) and can be downloaded through the Supplementary Information.
