# Supplementary material for: No evidence for environmental filtering of cavity‐nesting solitary bees and wasps by urbanization using trap nests
Source: Ecol Evol. 2022 Oct 1;12(10):e9360. doi: 10.1002/ece3.9360 (PMC9526028; doi:10.1002/ece3.9360)
Supplement: Supplementary file 1 — Appendix S1 [file ECE3-12-e9360-s001.docx]

**Table S1.** List of bee species, references for their identification, and the ecological traits used in the analysis. Body size was measured as intertegular span (ITS) on individuals except when too few samples were available and so value taken from Normandin et al. (2017). Six additional traits were obtained from the literature (superscript indicates reference): Origin (native or non-native), the nesting material type(s) used with the primary type (by amount) in **bold**, and the number of different types collected, dietary preference, taxonomic-level specialization (multi-order, single order, family), and numerical trophic rank (1^st^ = herbivore; all bees listed in this category).

| Family | Species | ID Ref | ITS | Origin | Nesting materials | | | Foraging materials | | |
| --- | --- | --- | --- | --- | --- | --- | --- | --- | --- | --- |
|  |  |  |  |  | Type | # Types | Diet | | Specialization | Rank |
| Apidae | *Anthophora terminalis Cresson* | A | 3.05^H^ | Native^I^ | Nest tube scrapings^K^ | Single | Pollen | | Multi-Order^I^ | 1^st^ |
| Megachilidae | *Anthidium manicatum* (Linnaeus) | B | 3.44 | Non-native^J^ | Leaf hair^J^ | Single | Pollen | | Multi-Order^I^ | 1^st^ |
|  | *Chelostoma rapunculi* (Lepeletier) | C | 1.66 | Non-native^G^ | Mud^D^ | Single | Pollen | | Family (Campanulaceae)^D^ | 1^st^ |
|  | *Chelostoma campanularum* (Kirby) | C | 1.11 | Non-native^C^ | Mud^N^ | Single | Pollen | | Family (Campanulaceae)^N^ | 1^st^ |
|  | *Heriades carinata* Cresson | A | 1.58 | Native^A^ | Resin^O^ | Single | Pollen | | Multi-Order^I^ | 1^st^ |
|  | *Heriades variolosa* (Cresson) | A | - | Native^A^ | **Resin** + Mud^P^ | Multi | Pollen | | Multi-Order^I^ | 1^st^ |
|  | *Hoplitis producta* (Cresson) | D | 2.10^H^ | Native^A^ | **Leaf pulp** + Sand^Q^ | Multi | Pollen | | Multi-Order^Q^ | 1^st^ |
|  | *Hoplitis spoliata* (Provancher) | D | 2.62 | Native^A^ | **Leaf pulp** + Pebbles^R^ | Multi | Pollen | | Multi-Order^I^ | 1^st^ |
|  | *Hoplitis truncata* (Cresson) | D | - | Native^A^ | Leaf pulp^S^ | Single | Pollen | | Multi-Order^I^ | 1^st^ |
|  | *Megachile brevis* Say | E | 2.93 | Native^E^ | Leaf cut^E^ | Single | Pollen | | Multi-Order^I^ | 1^st^ |
|  | *Megachile campanulae* (Robertson) | E | 2.42 | Native^E^ | Resin^M^ | Single | Pollen | | Multi-Order^I^ | 1^st^ |
|  | *Megachile centuncularis* (Linnaeus) | E | 3.05 | Non-native^L^ | Leaf cut^E^ | Single | Pollen | | Multi-Order^E^ | 1^st^ |
|  | *Megachile frigida* Smith | E | 3.41 | Native^E^ | Leaf cut^E^ | Single | Pollen | | Multi-Order^I^ | 1^st^ |
|  | *Megachile inermis* Provancher | E | 4.58 | Native^E^ | Leaf cut^E^ | Single | Pollen | | Multi-Order^I^ | 1^st^ |
|  | *Megachile mendica* Cresson | E | 3.40 | Native^E^ | Leaf cut^E^ | Single | Pollen | | Multi-Order^X^ | 1^st^ |
|  | *Megachile pugnata* Say | E | 3.45 | Native^E^ | **Leaf cut** + Pulp + Mud^T^ | Multi | Pollen | | Family (Asteraceae)^T^ | 1^st^ |
|  | *Megachile relativa* Cresson | E | 3.03 | Native^E^ | Leaf cut^E^ | Single | Pollen | | Multi-Order^I^ | 1^st^ |
|  | *Megachile rotundata* Fabricius | E | 2.48 | Non-native^G^ | Leaf cut^E^ | Single | Pollen | | Multi-Order^Y^ | 1^st^ |
|  | *Megachile sculpturalis* Smith | E | 4.05 | Non-native^G^ | Resin^U^ | Single | Pollen | | Multi-Order^Z^ | 1^st^ |
|  | *Osmia atriventris Cresson* | A | 2.33 | Native^A^ | Leaf pulp^V^ | Single | Pollen | | Multi-Order^I^ | 1^st^ |
|  | *Osmia caerulescens* (Linnaeus) | A | 2.56 | Non-native^G^ | Leaf pulp^G^ | Single | Pollen | | Multi-Order^I^ | 1^st^ |
|  | *Osmia lignaria* Say | A | 3.65 | Native^A^ | Mud^V^ | Single | Pollen | | Multi-Order^I^ | 1^st^ |
|  | *Osmia pumila* Cresson | A | 1.95 | Native^A^ | Leaf pulp^V^ | Single | Pollen | | Multi-Order^I^ | 1^st^ |
| Colletidae | *Hylaeus affinis* (Smith) | F | 1.33 | Native^F^ | Secretions^W^ | None | Pollen | | Multi-Order^I^ | 1^st^ |
|  | *Hylaeus annulatus* (Linnaeus) | F | 1.23 | Native^I^ | Secretions^W^ | None | Pollen | | Multi-Order^I^ | 1^st^ |
|  | *Hylaeus hyalinatus* Smith | G | 1.71^H^ | Non-native^G^ | Secretions^W^ | None | Pollen | | Multi-Order^I^ | 1^st^ |
|  | *Hylaeus leptocephalus* (Morawitz) | F | 1.11 | Non-native^G^ | Secretions^W^ | None | Pollen | | Multi-Order^I^ | 1^st^ |
|  | *Hylaeus mesillae* (Cockerell) | F | 1.60 | Native^F^ | Secretions^W^ | None | Pollen | | Multi-Order^I^ | 1^st^ |
|  | *Hylaeus modestus* Say | F | 1.53 | Native^F^ | Secretions^W^ | None | Pollen | | Multi-Order^I^ | 1^st^ |
|  | *Hylaeus punctatus (*Brullé) | G | 1.23^H^ | Non-native^G^ | Secretions^W^ | None | Pollen | | Multi-Order^I^ | 1^st^ |
|  | *Hylaeus verticalis* (Cresson) | F | 1.30 | Native^F^ | Secretions^W^ | None | Pollen | | Multi-Order^I^ | 1^st^ |

**Nesting material type:** ‘mud’ was used as a synonym for cases where literature refers to ‘soil’. *Anthophora terminalis* scraped the inner tunnel of the cardboard nesting tube to create brood cell partitions, similar to their behaviour in sumac stems and other natural nesting materials (Medler, 1964).

**Foraging material specialization:** *C. rapunculi* and *C. campanularum* are pollen specialists on the genus *Campanula* but known to sometimes visit other related genera and especially for nectar, and so specialization is listed at the family level.

| Family | Species | ID Ref | ITS | Origin | Nesting materials | | Foraging materials | | |
| --- | --- | --- | --- | --- | --- | --- | --- | --- | --- |
|  |  |  |  |  | Types | # Types | Diet | Specialization | Rank |
| Vespidae | *Ancistrocerus adiabatus* (Saussure) | AA | 2.05 | Native^AA^ | Mud^AA^ | Single | Caterpillars^AJ^ | Order (Lepidoptera)^AJ^ | 2^nd^ |
|  | *Ancistrocerus antilope* Panzer | AA | 2.62 | Native^AJ^ | Mud^AA^ | Single | Caterpillars^AJ^ | Order (Lepidoptera)^AJ^ | 2^nd^ |
|  | *Ancistrocerus gazella* (Panzer) | AA | 2.12 | Non-native^C^ | Mud^AL^ | Single | Caterpillars^AL^ | Order (Lepidoptera)^AL^ | 2^nd^ |
|  | *Euodynerus foraminatus* (Saussure) | AA | 2.75 | Native^AK^ | Mud^AA^ | Single | Caterpillars^AJ^ | Order (Lepidoptera)^AJ^ | 2^nd^ |
|  | *Euodynerus pantitarsis* (Bohart) | AA | 3.02 | Native^AK^ | Mud^AK^ | Single | Caterpillars^AK^ | Order (Lepidoptera)^AK^ | 2^nd^ |
|  | *Monobia quadridrens* (Linnaeus) | AA | 2.90 | Native^AK^ | Mud^AJ^ | Single | Caterpillars^AJ^ | Order (Lepidoptera)^AJ^ | 2^nd^ |
|  | *Symmorphus albomarginatus* (Saussure) | AA, AB | 1.50 | Native^AB^ | Mud^AJ^ | Single | Beetle larva^AJ^ | Family (Chrysomelidae)^AJ^ | 2^nd^ |
|  | *Symmorphus bifasciatus* (Linnaeus) | AC | - | Non-native^AL^ | Mud^AL^ | Single | Beetle larva^AL^ | Family (Chrysomelidae)^AJ^ | 2^nd^ |
|  | *Symmorphus canadensis* (Saussure) | AA, AB | 1.42 | Native^AB^ | Mud^AJ^ | Single | **Beetle larva**+ Caterpillars^AJ^ | Multi-Order (Coleoptera, Lepidoptera)^AJ^ | 2^nd^ |
|  | *Symmorphus cristatus* (Saussure) | AA, AB | 1.63 | Native^AB^ | Mud^AJ^ | Single | Beetle larva^AJ^ | Family (Chrysomelidae)^AJ^ | 2^nd^ |
| Pompilidae | *Auplopus mellipes* (Say) | AD | 1.40 | Native^AD^ | Mud^AK^ | Single | Single spider^AK^ | Order (Araneae)^AK^ | 3^rd^ |
|  | *Dipogon sayi* Banks | AD | 1.31 | Native^AM^ | **Mud** +  Leaf pulp^AM^ | Multi | Single spider^AM^ | Order (Araneae)^AM^ | 3^rd^ |
| Sphecidae | *Isodontia mexicana* (Saussure) | AE | 2.56 | Native^AE^ | Grass^AE^ | Single | Tree crickets^AE^ | Order (Orthoptera)^AE^ | 2^nd^ |
| Crabronidae | *Passaloecus cuspidatus* F. Smith | AF | 1.15 | Native^AF^ | Resin^AF^ | Single | Aphids^AF^ | Family (Aphididae)^AF^ | 2^nd^ |
|  | *Passaloecus gracilis* (Curtis) | AF | 1.11 | Non-native^AK^ | Resin^AL^ | Single | Aphids^AL^ | Family (Aphididae^AL^ | 2^nd^ |
|  | *Passaloecus monilicornis* Dahlbom | AF | - | Native^AF^ | Resin^AF^ | Single | Aphids^AF^ | Family (Aphididae)^AF^ | 2^nd^ |
|  | *Psenulus pallipes* (Panzer) | AG, AH | 1.03 | Non-native^AH^ | Secretions^AL^ | None | Aphids^AL^ | Family (Aphididae^AL^ | 2^nd^ |
|  | *Trypoxylon collinum* F. Smith | AI | 1.87 | Native^AI^ | Mud^AI^ | Single | Spiders^AI^ | Order (Araneae)^AI^ | 3^rd^ |
|  | *Trypoxylon frigidum* F. Smith | AI | 1.03 | Native^AI^ | Mud^AI^ | Single | Spiders^AI^ | Order (Araneae)^AI^ | 3^rd^ |
|  | *Trypoxylon lactitarse* Saussure | U | 3.30 | Native^AI^ | Mud^AI^ | Single | Spiders^AI^ | Order (Araneae)^AI^ | 3^rd^ |

**Table S2.** List of wasp species, references for their identification, and the ecological traits used in the analysis. Body size was measured as intertegular span (ITS) in mm. Six additional traits were obtained from the literature (superscript indicates reference): Origin (native or non-native), the nesting material type(s) used, and the number of different types collected, dietary preference, taxonomic-level specialization (multi-order, single order, family), and numerical trophic rank (1^st^ = herbivore, 2^nd^ = feeds on herbivores, 3^rd^ = feeds on carnivores).

### References (for Table S1 and Table S2)

### ^A.^ Mitchell, T.B. (1962) Bees of the Eastern United States. II. Technical bulletin (North Carolina Agricultural Experiment Station) 152, 1-557.

### ^B.^ Miller, S.R., Gaebel, R., Mitchell, R.J. & Arduser, M. (2002) Occurrence of two species of Old World bees, *Anthidium manicatum* and *A. oblongatum* (Apoidea: Megachilidae), in northern Ohio and southern Michigan. *The Great Lakes Entomologist* 35, 12.

### ^C.^ Buck, M., Paiero, S.M. & Marshall, S.A. (2006) New records of native and introduced aculeate Hymenoptera from Ontario, with keys to eastern Canadian species of *Cerceris* (Crabronidae) and eastern Nearctic species of *Chelostoma*(Megachilidae). *Journal of the Entomological Society of* Ontario 136, 37–52.

### ^D.^ Müller, A. (2015) Palaearctic *Chelostoma* bees of the subgenus *Gyrodromella* (Megachilidae, Osmiini): Biology, taxonomy and key to species. *Zootaxa* 3936, 408-420.

### ^E.^ Sheffield, C.S., Ratti, C., Packer, L. & Griswold, T. (2011) Leafcutter and mason bees of the genus *Megachile* Latreille (Hymenoptera: Megachilidae) in Canada and Alaska. *Canadian Journal of Arthropod Identification*18, 1-107. doi:10.17161/jom.v0i69.6532.

### ^F.^ Mitchell, T.B. (1960) Bees of the Eastern United States. I. North Carolina Agricultural Experimental Station Technical Bulletin 141, 1–538.

### ^G.^ Sheffield, C.S., Dumesh, S. & Cheryomina, M. (2011) *Hylaeus punctatus* (Hymenoptera: Colletidae), a bee species new to Canada, with notes on other non-native species. *Journal of the Entomological Society of Ontario* 142, 29-43.

### ^H.^ Normandin, É., Vereecken, N.J., Buddle, C.M. & Fournier, V. (2017) Taxonomic and functional trait diversity of wild bees in different urban settings. *PeerJ*5, e3051.

### ^I.^ Ascher, J.S., & Pickering, J. (2020) Discover Life bee species guide and world checklist (Hymenoptera: Apoidea: Anthophila). Available at: <https://www.discoverlife.org/20/q?search=Apoidea>

### ^J.^ Gibbs, J. & Sheffield, C.S. (2009) Rapid range expansion of the wool-carder bee, *Anthidium manicatum* (Linnaeus)(Hymenoptera: Megachilidae), in North America. *Journal of the Kansas Entomological Society*82, 21-29.

^K^ Medler, J. T. (1964). *Anthophora* (Clisodon) *terminalis* Cresson in trap-nests in Wisconsin (Hymenoptera: Anthophoridae). *The Canadian Entomologist* 96, 1332-1336

**^L.^** Giles, V. & Ascher, J.S. (2006). A survey of the bees of the Black Rock Forest Preserve, New York (Hymenoptera: Apoidea). *Journal of Hymenoptera Research* 15, 208– 231.

### ^M.^ Michener (2007) The Bees of the World. 2^nd^ Edition. John Hopkins University Press, Maryland, USA.

### ^N.^ Eickwort, G.C. (1980) Two European species of *Chelostoma* established in New York State (Hymenoptera: Megachilidae). *Psyche*, 87, 315-323.

### ^O.^ Matthews, R.W. (1965) The biology of *Heriades carinata* Cresson (Hymenoptera, Megachilidae). *Contributions of the American Entomological Institute* 1, 1–33

### ^P.^ Fischer, R.L. (1955) A nest of *Heriades variolosus* (Cress.) (Hymenoptera: Megachilidae). *The Canadian Entomologist* 87, 33–36. doi:10.4039/ent8733-1.

### ^Q.^ Medler, J.T. (1961) A Note on *Hoplitis producta* (Cress.) in Wisconsin (Hymenoptera: Megachilidae). *The Canadian Entomologist* 93, 571–573. doi:10.4039/ent93571-7

### ^R. A^Medler, J.T. (1967) *Hoplitis cylindrica* in trap nests in Wisconsin (Hymenoptera: Megachilidae). *Journal of the Kansas Entomological Society* 40, 137-140.

### ^S.^ Michener, C.D. (1947) A revision of the American species of *Hoplitis* (Hymenoptera, Megachilidae). *Bulletin of the American Museum of Natural History* 89, 1-68.

### ^T.^ Frolich, D.R. & Parker, F.D. (1983) Nest building behavior and development of the sunflower leafcutter bee: *Eumegachile* (Sayapis) *pugnata* (Say)(Hymenoptera: Megachilidae). *Psyche* 90, 193-209.

### ^U.^ Batra, S. W. (1998). Biology of the Giant Resin Bee, *Megachile sculpturalis* Smith, a Conspicuos New Immigrant in Maryland. *The Maryland Naturalist* 42, 1.

### ^V.^ Cane, J.H., Griswold, T. & Parker, F.D. (2007) Substrates and materials used for nesting by North American *Osmia* bees (Hymenoptera: Apiformes: Megachilidae). *Annals of the Entomological Society of* America 100, 350-358.

### ^W.^ Batra, S.W. (1972). Some properties of the nest-building secretions of *Nomia*, *Anthophora*, *Hylaeus* and other bees. *Journal of the Kansas Entomological Society* 45, 208-218.

### ^X.^ Bzdyk, E.L. (2012) A revision of the *Megachile* subgenus Litomegachile Mitchell with an illustrated key and description of a new species (Hymenoptera, Megachilidae, Megachilini). *ZooKeys* 221, 31-61. doi:10.3897/zookeys.221.3234

### ^Y.^ Pitts-Singer, T.L. & Cane, J.H. (2011) The Alfalfa Leafcutting Bee, *Megachile rotundata*: The world’s most intensively managed solitary bee. *Annual Review of Entomology* 56, 221–237.

### ^Z.^ Mangum, W. A., & Brooks, R. W. (1997) First records of Megachile (Callomegachile) sculpturalis Smith (Hymenoptera: Megachilidae) in the continental United States. *Journal of the Kansas Entomological Society* 70, 140-142.

### ^AA.^ Buck, M., Marshall, S.A. & Cheung, D.K. (2008) Identification Atlas of the Vespidae (Hymenoptera, Aculeata) of the northeastern Nearctic region. *Canadian Journal of Arthropod Identification* 5, 1-492.

### ^AB.^ Cumming, J.M. (1989) Classification and evolution of the eumenine wasp genus *Symmorphus* Wesmael (Hymenoptera: Vespidae). *Memoirs of the Entomological Society of Canada* 148, 168 pp.

### ^AC.^ Gittins, A.R. (1969) Revision of the Nearctic Psenini (Hymenoptera: Sphecidae) I. Redescriptions and keys to the genera and subgenera. *Transactions of the American Entomological Society* 95, 49-76.

### ^AD.^ Townes, H. (1957) Nearctic wasps of the subfamilies Pepsinae and Ceropalinae. *Bulletin of the United States National Museum*. 1-286. <https://doi.org/10.5479/si.03629236.209.1>

### ^AE.^ Bohart, R.M., Bohart, R.M. & Menke, A.S. (1976) *Sphecid Wasps of the World: A Generic Revision*. University of California Press.

### ^AF.^ Vincent, D.L. (1978) A revision of the genus *Passaloecus* (Hymenoptera: Sphecidae) in America North of Mexico. *Wasmann Journal of Biology* 36: 127-198.

### ^AG.^ Kim, J.K. & Lee, S.G. (2006) Taxonomic review of the genus *Symmorphus* Wesmael (Hymenoptera: Vespidae: Eumeninae) from the Far East. *Entomological Research* 36, 27-41.

### ^AH.^ Schmid-Egger, C. (2016) The *Psenulus pallipes* species group in Central Europe (Hymenoptera, Crabronidae). *Ampulex* 8, 40-44.

### ^AI.^ Sandhouse, G.A. (1940) A review of the Nearctic wasps of the genus *Trypoxylon* (Hymenoptera: Sphecidae). *The American Midland Naturalist* 24, 133-176.

### ^AJ.^ Krombein, K.V. (1967) Trap-Nesting Wasps and Bees: Life Histories, Nests, and Associates. Smithsonian Press, Washington D.C., 570 pp.

### ^AK.^ Krombein, K.V. (1979) Superfamily Vespoidea. *In* Catalog of Hymenoptera in America north of Mexico Vol. 2, Apocrita (Aculeata). *Edited by* K.V. Krombein, P.D. Hurd, D.R. Smith, and B.D. Burks. Smithsonian Institution Press, Washington, 1469–1522.

### ^AL.^ Budrys, E., Budriene, A. & Orlovskyte, S. (2014) SCALES: Wasps and Bees Database. http://scales.ckff.si/scaletool/Available at: <http://scales.ckff.si/scaletool/>.

### ^AM.^ Medler, J.T. & Koerber, T.W. (1957). Biology of *Dipogon sayi* Banks (Hymenoptera, Pompilidae) in trap-nests in Wisconsin. *Annals of the Entomological Society of America* 50, 621-625.

**Table S3**. Generalized **v**ariance inflation factors GVIF) for linear regression models with a set of different predictors at the 250m spatial scale. Using the *vif* function in the “car” R package, GVIFs are calculated if the linear model has more than 1 df (e.g, due to inclusion of categorical variables). Here, GVIFs can be interpreted as the inflation in size of the confidence ellipse or ellipsoid for the coefficients of the term in comparison with what would be obtained for orthogonal data, where GVIF > 5 indicate collinearity.

| Model 1: ses.MFD ~ percent tree cover + percent grass cover + percent impervious surface + urban green space type | | | |
| --- | --- | --- | --- |
|  | **GVIF** | **Df** | **GVIF^(1/(2*Df))^** |
| Percent closed green cover (250 m) | 3.50 | 1 | 1.88 |
| Percent open green cover (250 m) | 9.96 | 1 | 3.16 |
| Percent impervious surface (250 m) | 10.40 | 1 | 3.23 |
| Urban green space type | 1.93 | 3 | 1.16 |
| Model 2: ses.MFD ~ percent grass cover + percent impervious surface + urban green space type | | | |
|  | **GVIF** | **Df** | **GVIF^(1/(2*Df))^** |
| Percent open green cover (250 m) | 1.21 | 1 | 1.10 |
| Percent impervious surface (250 m) | 1.62 | 1 | 1.27 |
| Urban green space type | 1.74 | 3 | 1.10 |

### Table S4. Generalized variance inflation factors GVIF) for linear regression models with a set of different predictors at the 500m spatial scale. Using the *vif* function in the “car” R package, GVIFs are calculated if the linear model has more than 1 df (e.g, due to inclusion of categorical variables). Here, GVIFs can be interpreted as the inflation in size of the confidence ellipse or ellipsoid for the coefficients of the term in comparison with what would be obtained for orthogonal data, where GVIF > 5 indicate collinearity.

| Formula: ses.MFD ~ percent tree cover + percent grass cover + percent impervious surface + urban green space type | | | |
| --- | --- | --- | --- |
|  | **GVIF** | **Df** | **GVIF^(1/(2*Df))^** |
| Percent closed green cover (500 m) | 6.21 | 1 | 2.49 |
| Percent open green cover (500 m) | 25.24 | 1 | 5.02 |
| Percent impervious surface (500 m) | 28.34 | 1 | 5.32 |
| Urban green space type | 1.56 | 3 | 1.08 |
| Formula: ses.MFD ~ percent grass cover + percent impervious surface + urban green space type | | | |
|  | **GVIF** | **Df** | **GVIF^(1/(2*Df))^** |
| Percent open green cover (500 m) | 1.16 | 1 | 1.08 |
| Percent impervious surface (500 m) | 1.53 | 1 | 1.24 |
| Urban green space type | 1.51 | 3 | 1.07 |

**Table S5**. Regression output on the relationship between two land cover classes (*i.e.*, percent open green cover, percent impervious surface), urban green space type (*i.e.*, community garden, home garden, public park, and green roof) and ses.MFD at the 250m scale from 136 sites (df = 130 ). Here, ‘community garden’ was used as the reference level for urban green space type.

|  | Estimate | Std. Error | *t* value | p-value |
| --- | --- | --- | --- | --- |
| Intercept | 0.277 | 0.518 | 0.536 | 0.59 |
| Percent open green | -0.013 | 0.012 | -1.080 | 0.28 |
| Percent impervious surface | 0.003 | 0.005 | -0.576 | 0.57 |
| Home garden | 0.055 | 0.333 | -0.165 | 0.87 |
| Public park | -0.179 | 0.367 | -0.488 | 0.63 |
| Green roof | 1.233 | 0.434 | -2.842 | <0.01 |

### Table S6. Regression output on the relationship between two land cover classes (*i.e.*, percent open green cover, percent impervious surface), urban green space type (*i.e.*, community garden, home garden, public park, and green roof) and ses.MFD at the 500m scale from 136 sites (df = 130). Here, ‘community garden’ was used as the reference level for urban green space type.

|  | Estimate | Std. Error | *t* value | p-value |
| --- | --- | --- | --- | --- |
| Intercept | 0.650 | 0.558 | 1.166 | 0.25 |
| Percent open green | -0.020 | 0.014 | -1.403 | 0.16 |
| Percent impervious surface | -0.008 | 0.006 | -1.393 | 0.17 |
| Home garden | -0.043 | 0.322 | -0.134 | 0.89 |
| Public park | -0.232 | 0.356 | -0.652 | 0.52 |
| Green roof | -1.174 | 0.416 | -2.825 | <0.01 |


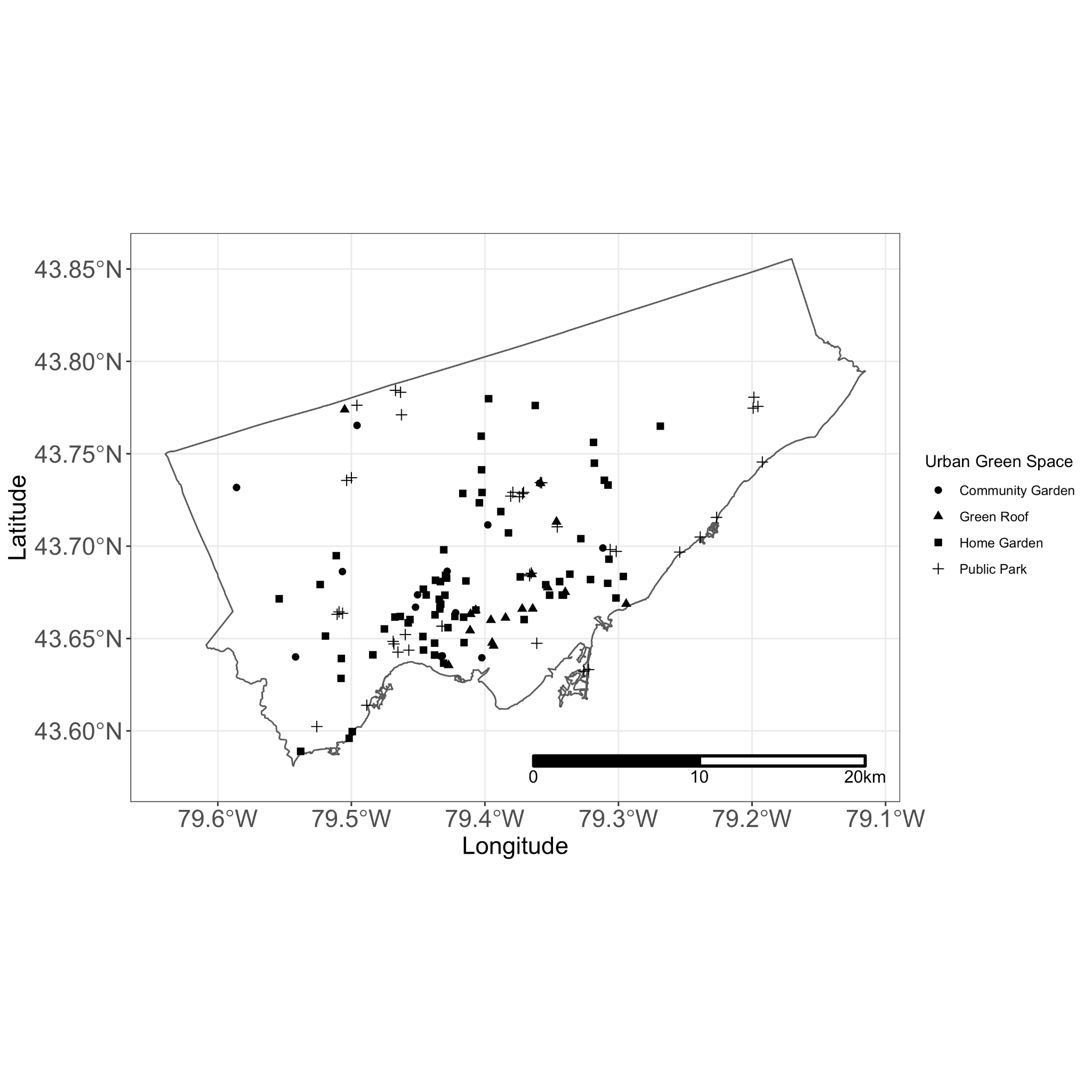
**Figure S1.** The spatial distribution of the sampled urban green space sites (i.e., community gardens, home gardens, green roofs, and public parks). The source material for the regional municipal boundary is from the City of Toronto open data portal. The geographic coordinate system is WGS84 (latitude and longitude).

###
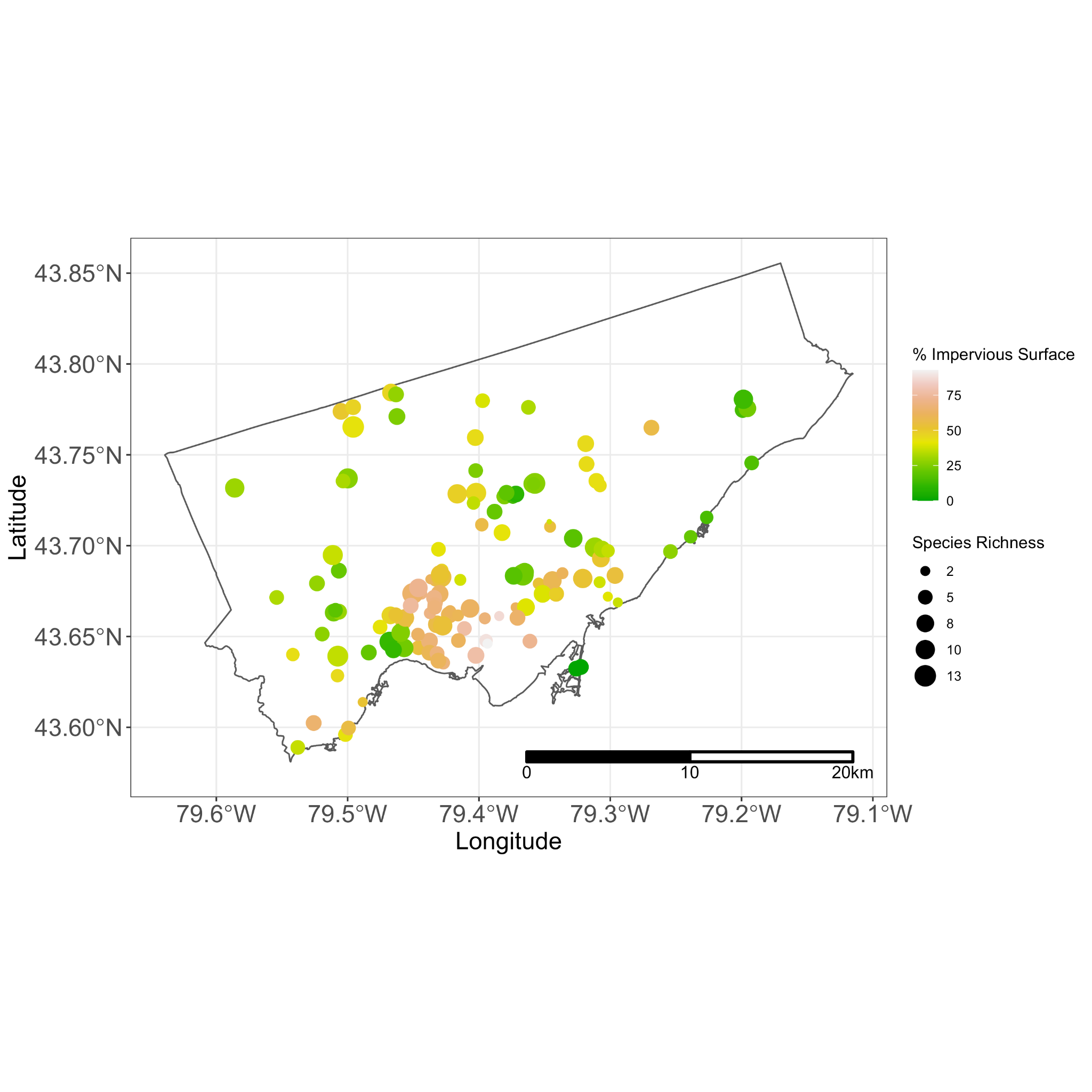
 Figure S2. Map of an urbanization (impervious surface) gradient of all sampled sites at the 500 m buffer radii. The size of the circles represents different levels of species richness. The source material for the regional municipal boundary is from the City of Toronto open data portal. The geographic coordinate system is WGS84 (latitude and longitude).


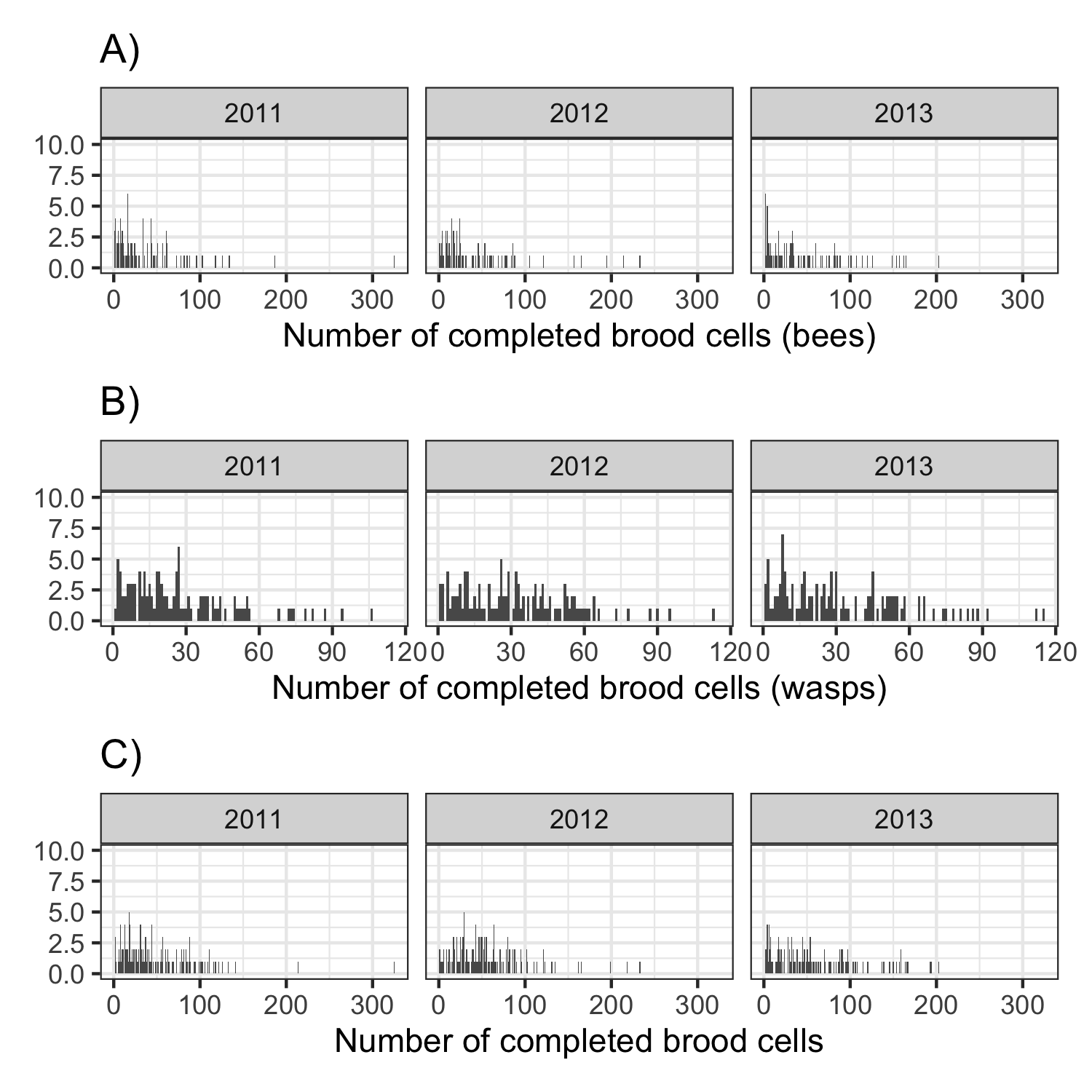


### Figure S3. Number of completed brood cells, at the site-level, for A) cavity-nesting bees and B) cavity-nesting wasps, and C) both bees and wasps across 140 sites for three consecutive sampling years (2011-2013).

###
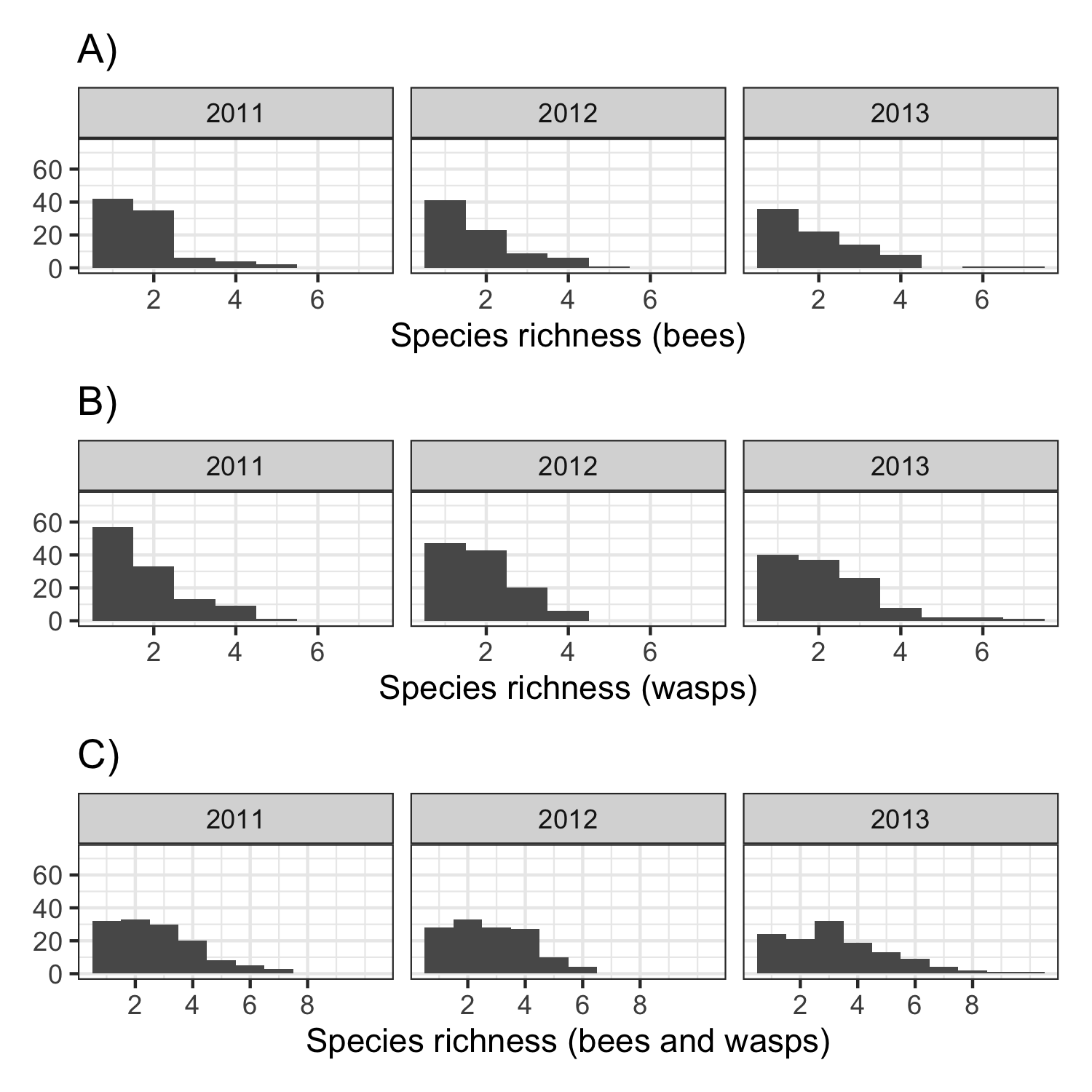


### Figure S4. Species richness from A) cavity-nesting bees, B) cavity-nesting wasps and C) both bees and wasps across 140 sites for three consecutive sampling years (2011-2013).


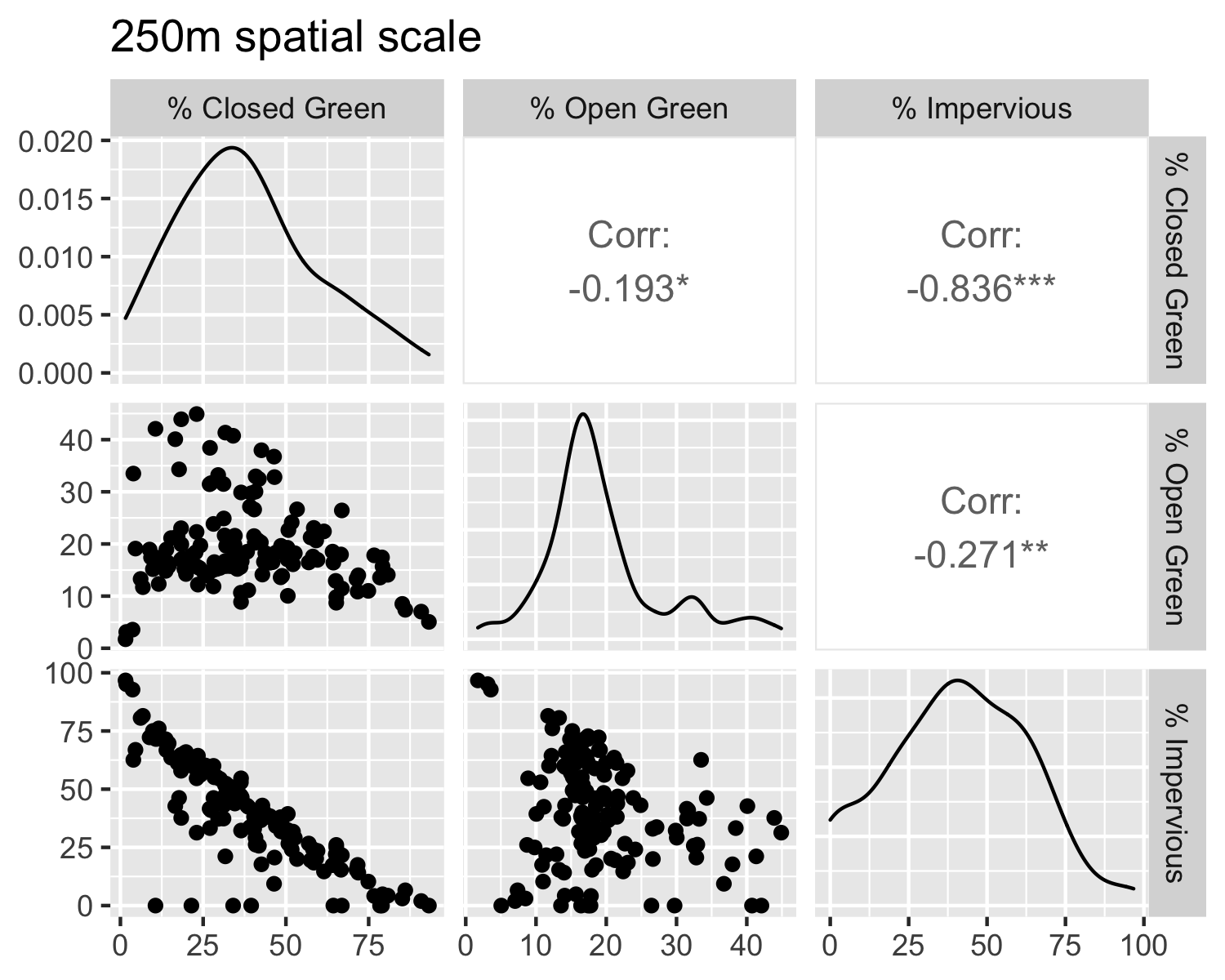


### Figure S5. The relationships between the three land cover classes at the 250 m scale for 140 sites. Diagonal elements represent the kernel density estimation for each variable, lower diagonal elements show scatter plots relationships between two variables, and the upper diagonal elements represent Pearson’ correlation coefficients. Symbols for statistical significance are as follows: p < 0.001 (***), p < 0.01 (**), p < 0.05 (*). Abbreviations are: “% closed green” represents percent closed green space, “% open green” represents percent open green space, and “% impervious” represents percent impervious surfaces.

###
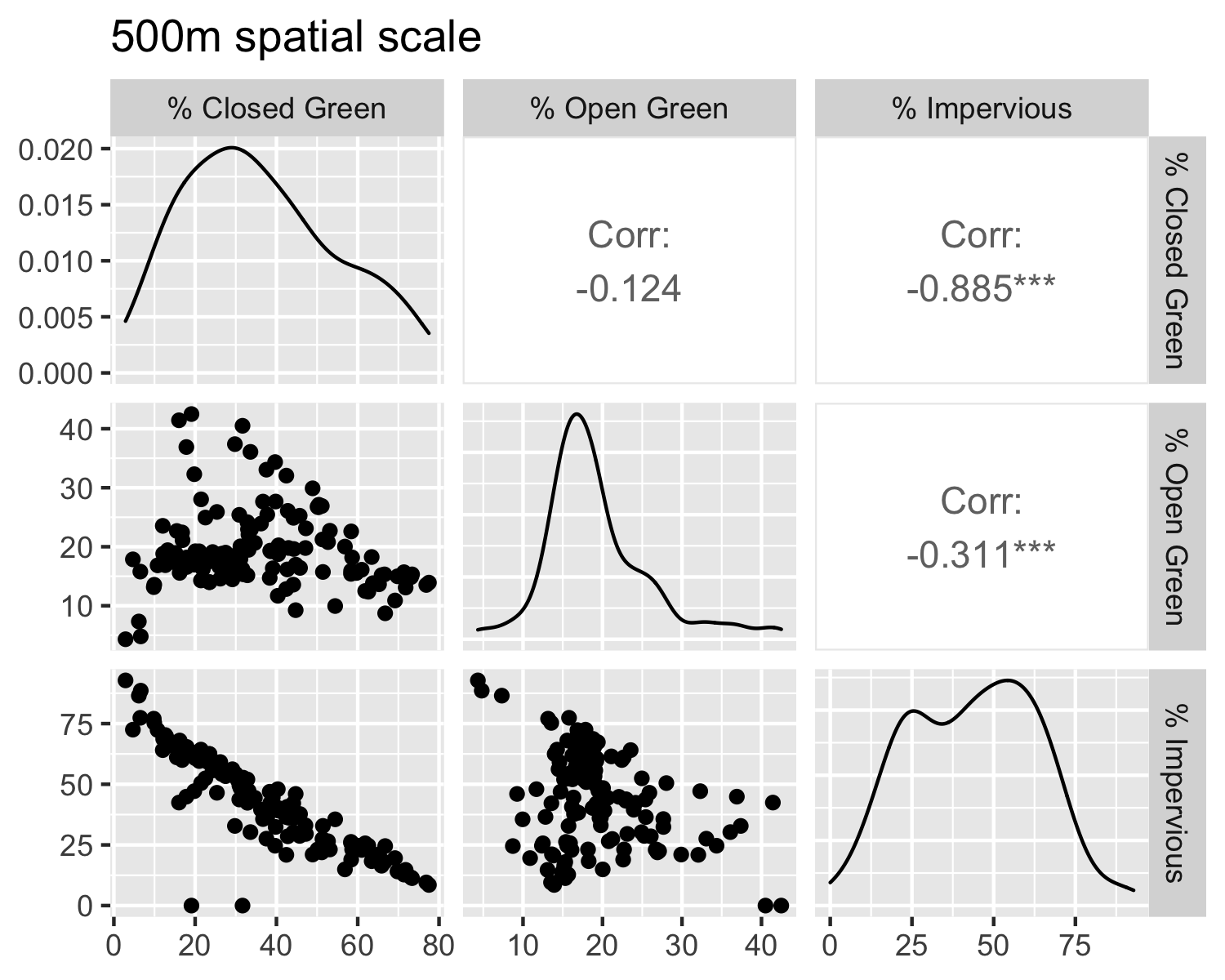


### Figure S6. The relationships between the three land cover classes at the 500m scale for 140 sites. Diagonal elements represent the kernel density estimation for each variable, lower diagonal elements show scatter plots relationships between two variables, and the upper diagonal elements represent Pearson’ correlation coefficients. Symbols for statistical significance are as follows: p < 0.001 (***), p < 0.01 (**), p < 0.05 (*). Abbreviations are: “% closed green” represents percent closed green space, “% open green” represents percent open green space, and “% impervious” represents percent impervious surfaces.

###

###
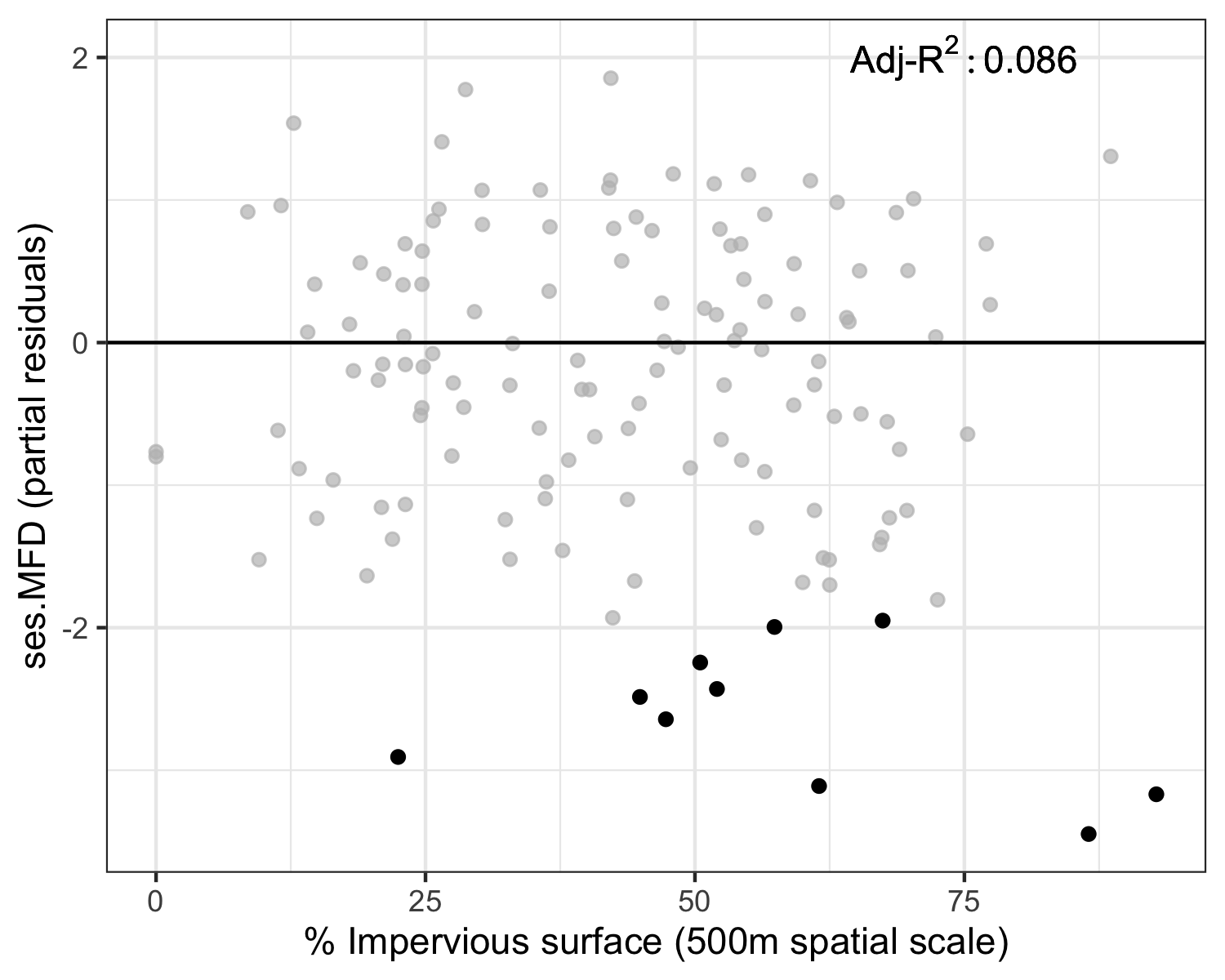


### Figure S7. A scatterplot of percent impervious surface and standardized mean pairwise functional distances at the 500m spatial scale. Highlighted black dots represent sites (n = 12) that meet CT criterion I - functionally clustered communities that are statistically significant (p < 0.05; see [Figure 3](#_heading=h.3rdcrjn)).


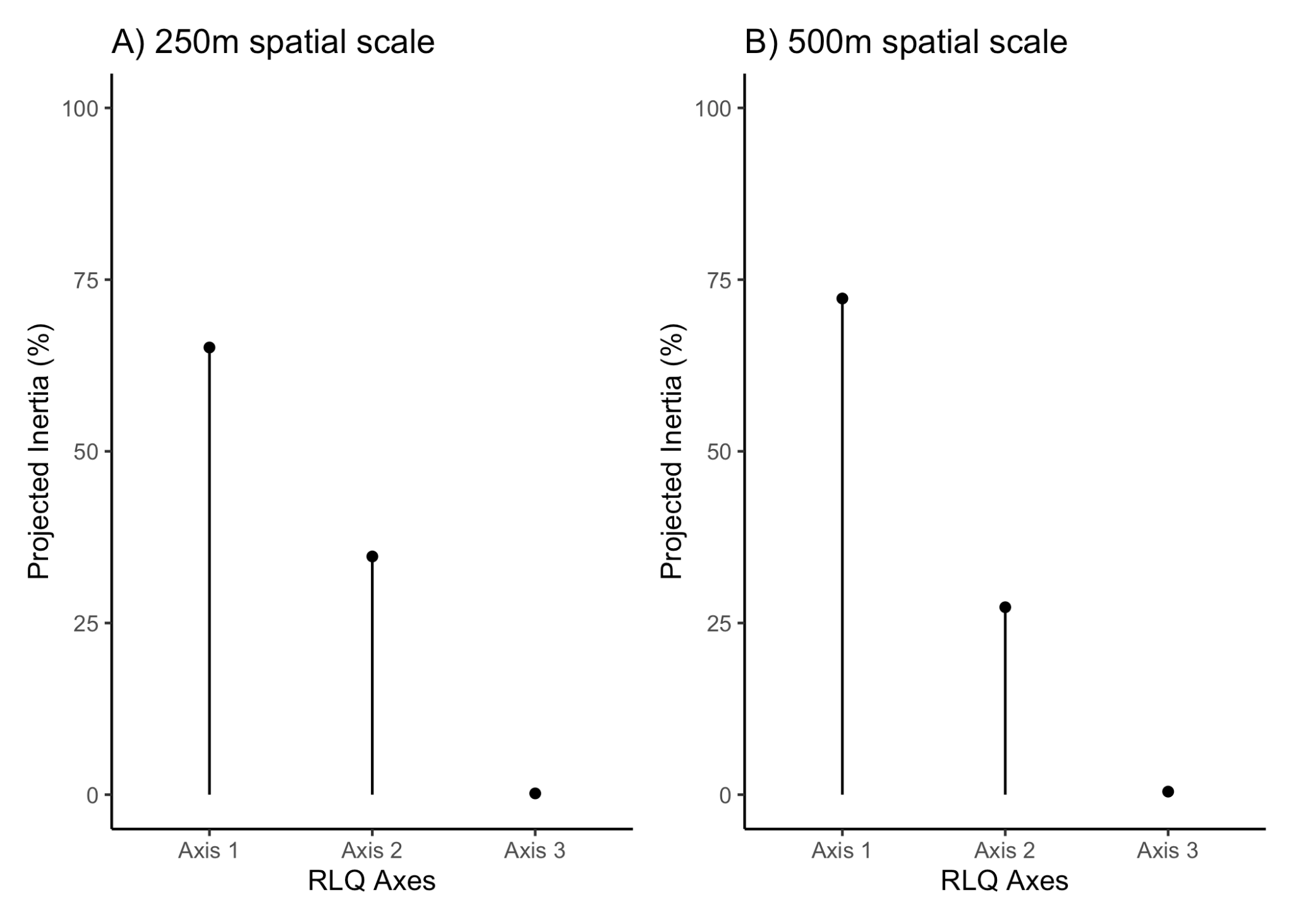


### Figure S8. Scree plot of the projected inertia for the three RLQ axes, which denotes the amount of variation explained per RLQ axis for each spatial scale (250m and 500m buffer radii).

###
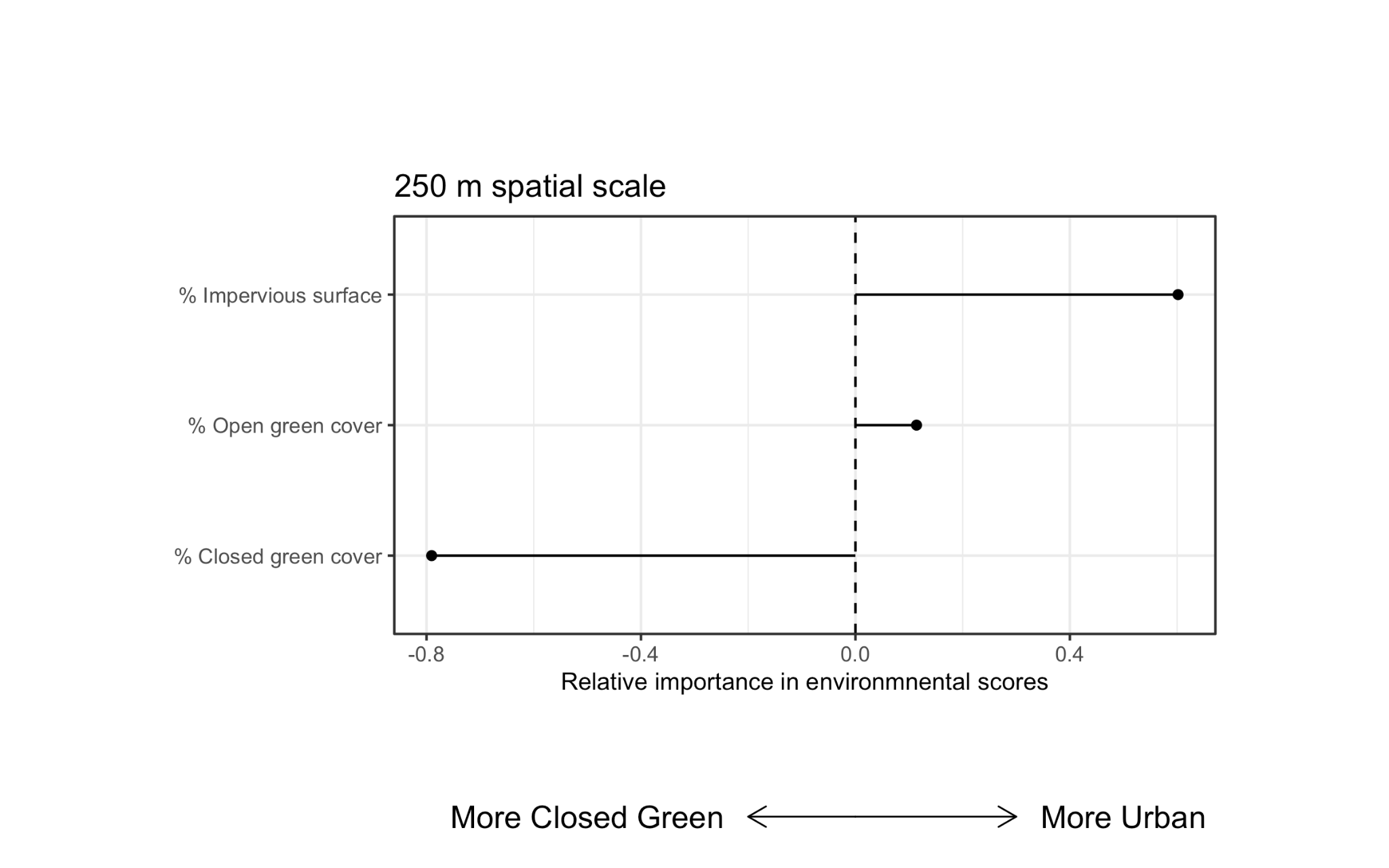
Figure S9. Relative importance of three environmental variables (i.e., percent impervious surface, percent open green cover, percent closed green cover) in RLQ axis 1 for 250m spatial scale. Note that the environmental scores are weighted by site scores from a correspondence analysis on the community data matrix.

###

###
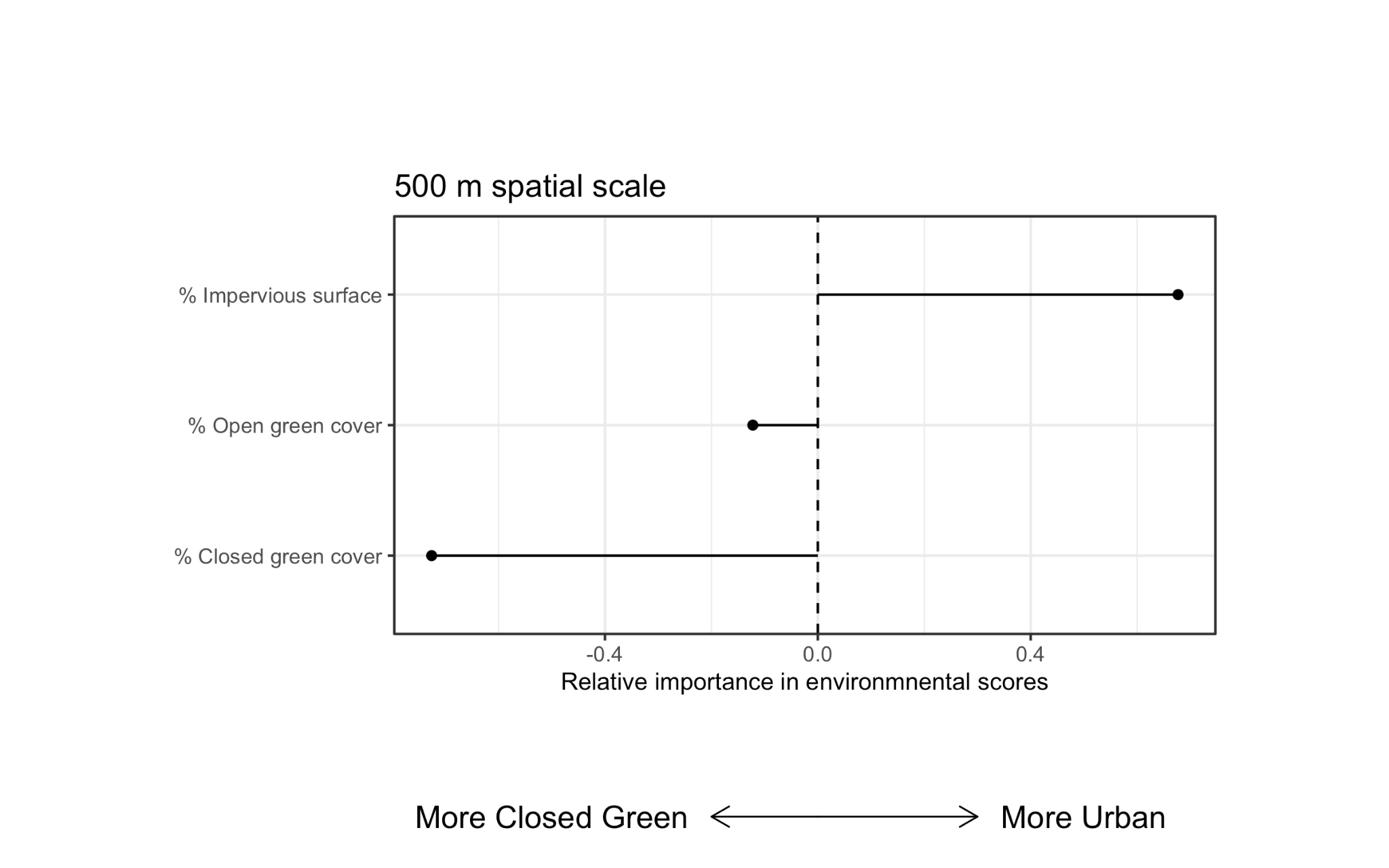
Figure S10. Relative importance of three environmental variables (i.e., percent impervious surface, percent open green cover, percent closed green cover) in RLQ axis 1 for 500m spatial scale. Note that the environmental scores are weighted by site scores from a correspondence analysis on the community data matrix.

###
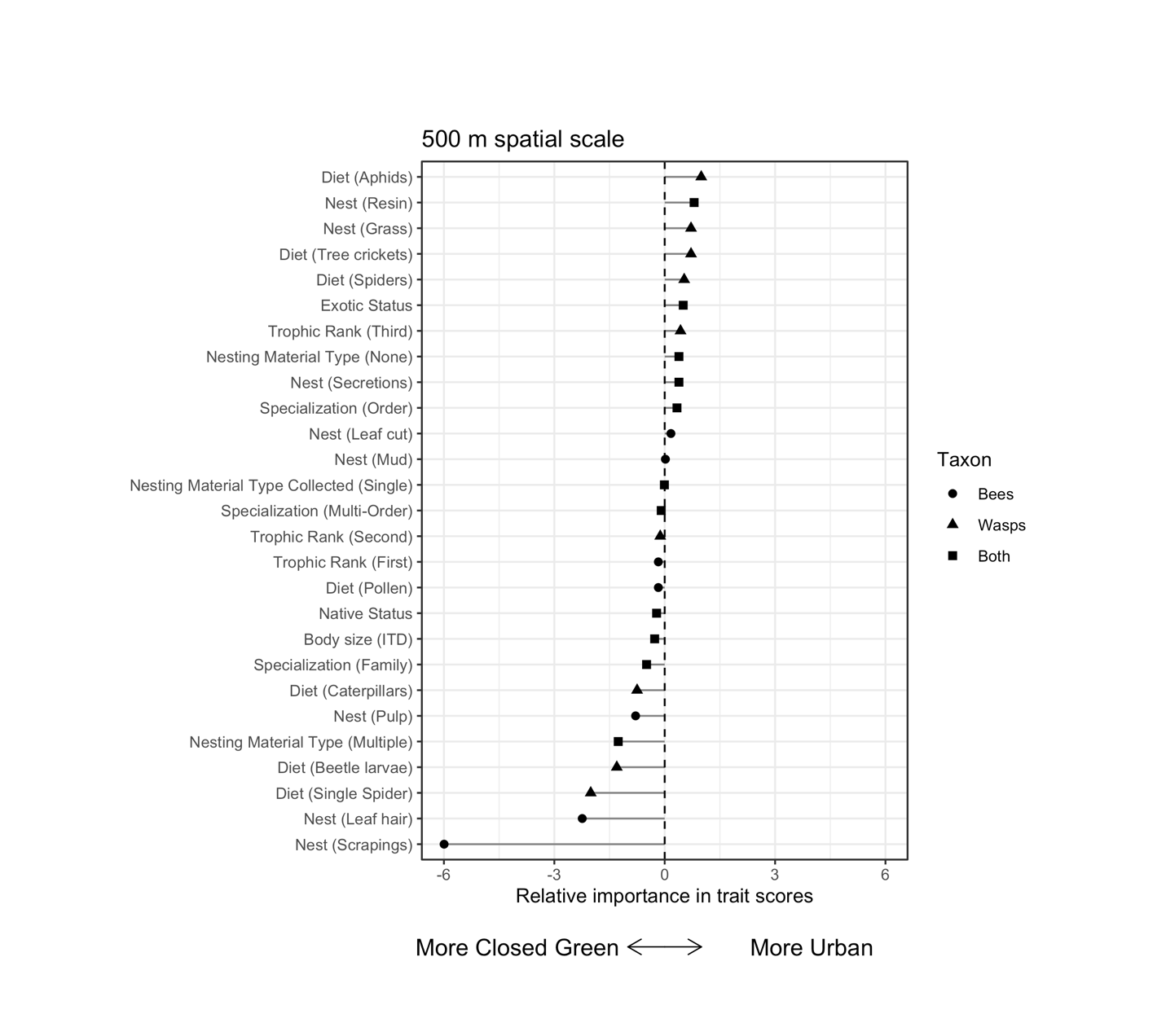


### Figure S11. The relative importance of trait scores from RLQ axis 1 for the 500m spatial scale. Negative score values indicate traits that are correlated with tree cover while positive score values indicate species that are correlated with impervious surfaces.

###
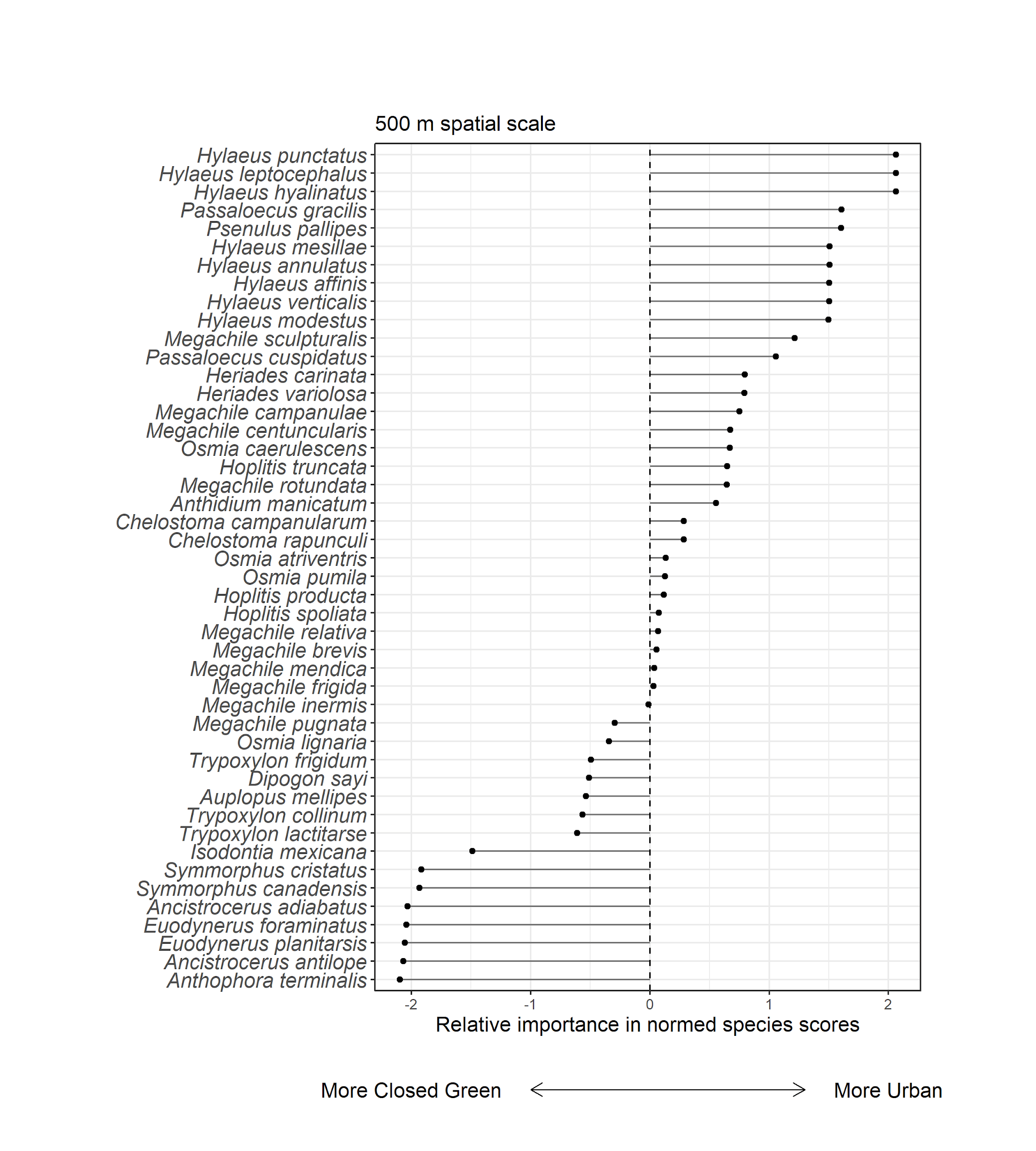


### Figure S12. The relative importance of normed species scores from RLQ axis 1 for the 500m spatial scale. Negative score values indicate species that inhabit sites with more closed green cover while positive score values indicate species that inhabit sites with more impervious surfaces.
